# Attachment Styles in Major Depressive Disorder: A Systematic Review and Meta‐Analysis

**DOI:** 10.1002/cpp.70343

**Published:** 2026-09-30

**Authors:** Lingfeng Xue, Virginia Chi Tong Chau, Anokhi Mehra, Rachel Woodham, Gabrielle Sheehan, Valentina Lorenzetti, Michal W. S. Ong, Muzzammil Hussain, Anna J. Lawes, Ramesh Muthuswamy, Cynthia H. Y. Fu

**Affiliations:** ^1^ Centre for Affective Disorders, Department of Psychological Medicine, Institute of Psychiatry, Psychology and Neuroscience King's College London London UK; ^2^ School of Psychology, Faculty of Health Sciences Australian Catholic University Melbourne Australia; ^3^ Imperial College Healthcare NHS Trust St Mary's Hospital London UK; ^4^ Department of Psychology University of East London London UK; ^5^ South London and Maudsley NHS Foundation Trust London UK; ^6^ National Institute for Health Research Biomedical Research Centre at South London and Maudsley NHS Foundation Trust King's College London London UK

**Keywords:** adult attachment, attachment anxiety and avoidance, insecure attachment, major depressive disorder, meta‐analysis, postpartum depression

## Abstract

Major depressive disorder (MDD), a leading cause of disability, is associated with difficulties in close relationships. Attachment theory links early caregiving to internal working models shaping emotion regulation, interpersonal functioning and depression vulnerability. Attachment patterns in clinically diagnosed MDD have not been synthesised. Following PRISMA (PROSPERO: CRD42017068580), we searched for studies of adults with standardised diagnoses of MDD or postpartum depression (PPD) and attachment measures. Random‐effects meta‐analyses expressed categorical outcomes as odds ratios (ORs) and continuous outcomes as Hedges' g. As a result, 61 studies met inclusion criteria: 33 case–control and 28 depression‐only, of which five provided qualitative data only. Categorically, MDD/PPD showed lower secure (OR = 0.20, 95% CI 0.15–0.26, *p* < 0.001) and higher anxious (OR = 2.46, 1.66–3.64, *p* < 0.001), avoidant (OR = 1.42, 1.04–1.95, *p* = 0.027) and fearful attachment (OR = 3.00, 1.97–4.54, *p* < 0.001) than healthy controls. On continuous measures, secure attachment was lower (*g* = −0.46, *p* = 0.003) and anxious (*g* = 0.63, *p* < 0.001) and avoidant (*g* = 0.41, *p* = 0.011) higher; fearful was not significant (*g* = 0.35, *p* = 0.062) until an outlier was removed. Single‐arm prevalence meta‐analyses found secure attachment in 24.1%, fearful in 26.9%, avoidant in 23.7% and anxious in 23.0%, with secure more prevalent in PPD than MDD (31.9% versus 21.3%, *p* = 0.009). In conclusion, MDD and PPD show lower secure and higher anxious, fearful and avoidant attachment than healthy controls. About three in four patients show insecure attachment, supporting attachment‐informed assessment and treatment planning.

## Summary

- Adults with major depressive disorder (MDD) or postpartum depression (PPD) show substantially lower secure attachment than healthy individuals, with about three in four having insecure attachment.
- Adults with MDD and postpartum depression show higher anxious, avoidant and fearful attachment than healthy individuals.
- Anxious attachment may contribute to interpersonal difficulties, including heightened concerns about rejection and abandonment and seeking reassurance and fearful attachment may present as negative expectations of both self and others, mistrust and conflicting needs for and fears of interpersonal closeness.
- Avoidant attachment may be clinically evident as minimisation of distress, withdrawal from relationships or difficulty engaging in treatment.
- Attachment style cannot be inferred from the diagnosis and assessment of attachment may help identify interpersonal vulnerabilities and support personalised case formulation and treatment planning.

## 1 Introduction

Major depressive disorder (MDD) is a leading cause of disability worldwide and carries profound personal and societal costs (GBD 2019 Mental Disorders Collaborators 2022). MDD is diagnosed on the basis of clinical symptoms, including persistent low mood or inability to experience pleasure accompanied by changes in sleep and appetite, low energy, impaired concentration and feelings of guilt (American Psychiatric Association 2013), yet it is also fundamentally an interpersonal disorder. Difficulties forming and maintaining supportive relationships are common and impaired social functioning predicts the onset, maintenance and relapse of depressive episodes (Kupferberg and Hasler 2023). Loneliness and social disconnection also prospectively increase the risk of MDD onset (Mann et al. 2022; Santini et al. 2020). These reciprocal processes, whereby depressive symptoms disrupt relational behaviour and relational difficulties in turn sustain depressive symptoms, position interpersonal dysfunction as both a core feature and a perpetuating mechanism in MDD.

Although genetic factors contribute to MDD, with heritability estimates of approximately 40%, environmental stressors have an important role in its development (Monroe and Simons 1991; Fernandez‐Pujals et al. 2015). Among environmental influences, early caregiving experiences have a central role in shaping interpersonal functioning and vulnerability to MDD (Bowlby 1969, 1973; Cummings and Cicchetti 1990). Attachment theory provides a developmental framework, linking early relationships with later emotional and interpersonal functioning (Bowlby 1969). Deeply embedded representations from pre‐verbal relational experiences shape models of the self and others that affect emotion regulation, interpersonal expectations and vulnerability to depressive illness (Perelberg 2017; Kupferberg and Hasler 2023).

Attachment theory proposes that early caregiving experiences give rise to internal working models of the self and others, cognitive‐affective representations of the self (e.g., as worthy or unworthy of care) and others (e.g., as responsive or unreliable) (Bowlby 1973, 1982; Bretherton 1991; Bretherton and Munholland 2008). These internal working models shape patterns of emotion regulation and interpersonal functioning throughout life, with attachment insecurity being associated with emotional and behavioural difficulties from childhood and adolescence (Pace et al. 2022). Consistent and sensitive caregiving promotes secure attachment, characterised by trust, effective emotion regulation, resilience and the capacity to explore the environment confidently (Ainsworth et al. 2015; Mikulincer et al. 1993). Conversely, inconsistent, rejecting or neglectful caregiving increases the likelihood of insecure attachment, characterised by negative models of the self (e.g., as unlovable), others (e.g., as unloving), or both (Ainsworth et al. 2015).

Adult attachment is commonly conceptualised using either dimensional or categorical models, with two continuous dimensions: attachment anxiety and avoidance, or four categorical styles: secure attachment, a form of insecure attachment namely avoidant/dismissive (minimising distress and reliance on others), anxious/preoccupied (maximising distress signals to maintain proximity), or fearful/disorganised (reflecting a collapse of strategy under threat) (Bartholomew and Horowitz 1991; Hesse and Main 2006; Main and Solomon 1990). These styles differ in how individuals regulate distress and seek interpersonal support. Prevalence estimates indicate that approximately 55% of adults exhibit secure attachment, 25% avoidant/dismissive and 20% anxious/preoccupied orientations (Shaver and Clark 1994).

Attachment theory provides a mechanistic framework for understanding the interpersonal disturbances that characterise MDD. Negative internal working models of the self and others can give rise to maladaptive interpersonal behaviours, including excessive reassurance seeking, withdrawal and emotional distancing, which can increase interpersonal conflict and reduce perceived social support (Kobak and Sceery 1988; Lakey and Orehek 2011; Williams and Riskind 2004). Individuals with MDD and insecure attachment not only perceive others as less available and less supportive but may also behave in ways that reinforce these expectations, leading to cycles of dissatisfaction in relationships and social isolation (Reis et al. 2017). As a result, interpersonal experiences become increasingly stressful and less rewarding, contributing to the onset and persistence of depressive symptoms (Cummings and Cicchetti 1990; Hames et al. 2013). Emotion regulation may mediate the relationship between insecure attachment and depressive symptoms, in particular through hyperactivating strategies, characteristic of anxious attachment, whereas evidence for deactivating strategies associated with avoidant attachment has been more mixed (Malik et al. 2015).

Previous meta‐analyses support an association between attachment patterns and depression, but have been limited in their measurement approach or study populations. Interview‐based syntheses using the Adult Attachment Interview have focused on categorical attachment classifications and combined non‐clinical, subclinical and clinical samples (Dagan et al. 2018). Self‐report meta‐analyses have examined continuous dimensions of attachment anxiety and avoidance, primarily in non‐clinical samples (Zheng et al. 2020), with subsequent work extending this dimensional approach to fearful/disorganised attachment, showing that attachment insecurity is associated with adult psychopathology more generally, including depression, anxiety and stress (Zhang et al. 2022) and similar associations in children and adolescents (Spruit et al. 2020). Similarly, in bipolar disorder, insecure attachment is associated with greater symptom burden (Suárez et al. 2026). Although these meta‐analyses report increased insecure attachment in depression (Dagan et al. 2018; Spruit et al. 2020; Zhang et al. 2022; Zheng et al. 2020), the included studies had not required a clinical diagnosis of MDD, had not integrated categorical and continuous attachment frameworks within a single synthesis and had not examined postpartum depression (PPD) separately.

PPD refers to a major depressive episode with a peripartum onset specifier rather than a distinct diagnostic disorder, thus sharing the same diagnostic threshold as non‐peripartum MDD. However, the postpartum period is characterised by unique biological and psychosocial changes, including abrupt hormonal withdrawal, the transition to parenthood and the formation of the mother‐infant relationship (Schiller et al. 2015). These changes may make attachment processes particularly salient as caregiving demands can reactivate an individual's own internal working models. Whether attachment patterns associated with non‐peripartum MDD generalise to PPD remains unclear, as previous meta‐analyses have not examined PPD separately. We thus included both MDD and PPD in the present review and conducted subgroup analyses to assess whether attachment patterns differ between peripartum and non‐peripartum depression.

In this systematic review and meta‐analysis, we examined attachment patterns in adults with MDD, including PPD, with diagnoses based on standardised clinical diagnostic criteria. We integrated categorical and continuous approaches as they reflect distinct but complementary models of attachment: typological model of Bartholomew and Horowitz (1991) and dimensional model of Brennan et al. (1998) that address related but non‐identical questions. Categorical analyses identify which attachment patterns are disproportionately represented, whereas continuous analyses quantify the extent to which individuals differ from healthy controls on attachment anxiety and avoidance. Our aims were threefold: (i) to compare attachment patterns between individuals with MDD/PPD and healthy controls across both categorical (typological) and continuous (dimensional) measurement frames, integrating findings across methodological traditions; (ii) to examine whether these patterns differ between non‐peripartum MDD and PPD through separate subgroup analyses; and (iii) to explore whether findings differed according to the attachment instrument used.

## 2 Methods

### 2.1 Literature Search

The review followed the Preferred Reporting Items for Systematic Reviews and Meta‐Analyses (PRISMA) guidelines (Page et al. 2021) and an a priori protocol was registered with the International Prospective Register of Systematic Reviews (PROSPERO) database (CRD42017068580). Publications were retrieved from PubMed, SCOPUS and APA PsycINFO databases from the start of the databases to 20 June 2026, applying the search terms ‘depression’ AND ‘attachment styles’. Additionally, the reference sections of relevant research articles were inspected for studies which may not have been identified via electronic database searches.

### 2.2 Inclusion and Exclusion Criteria

Inclusion criteria are as follows: 1) participants aged 18 years or above; 2) current or lifetime diagnosis of MDD; 3) diagnosis established using a standardised diagnostic instrument, such as the Structured Clinical Interview for DSM Disorders (SCID), Mini‐International Neuropsychiatric Interview (MINI) or clinical interview, operationalising Diagnostic and Statistical Manual of Mental Disorders (DSM) or International Classification of Diseases (ICD) criteria, or another well‐recognised diagnostic procedure (e.g., Research Diagnostic Criteria); 4) measurement of attachment styles with validated self‐report or clinician‐administered scales; and 5) written in English. Whereas, exclusion criteria are as follows: 1) commentaries, reports and single case studies; and 2) comorbid conditions. As MDD is frequently accompanied by comorbid diagnoses and participants' comorbidity information was not always reported in papers, the comorbidity assessment was applied pragmatically. As such, papers whose sampling frame noted a specific comorbidity, chronic medical illness, or severe psychiatric subtype (e.g., psychotic, bipolar or post‐traumatic stress disorders) were excluded as these conditions involve distinct etiological mechanisms and interpersonal features that could independently affect attachment styles (Degnan et al. 2022). Studies in which a minority of the MDD sample carried common secondary comorbid diagnoses, namely anxiety disorders, dysthymia, substance‐use and eating disorders, were included (Thaipisuttikul et al. 2014). Most studies had already excluded psychotic disorders, dementia, bipolar disorder and borderline personality disorder.

### 2.3 Study Selection

The study selection process comprised four phases: identification, screening, eligibility and inclusion. During the identification phase, the database search identified 6971 records, including 2877 from PubMed, 2118 from SCOPUS and 1976 from APA PsycINFO. After the removal of 2516 duplicate records, 4455 unique citations remained for screening. During the screening phase, titles and abstracts were independently assessed by reviewers (V.C., A.M., A.L., M.O., G.S., R.W. and G.S.), with disagreements resolved through discussion with an additional reviewer (C.F.) until consensus was reached. An audit trail documenting all screening decisions was maintained throughout the selection process to ensure transparency and reproducibility. During the eligibility phase, 597 articles were assessed in full text against the predefined inclusion and exclusion criteria and the reference lists of relevant studies were manually searched to identify additional eligible records. Finally, during the inclusion phase, 61 studies met the eligibility criteria and were included in the systematic review, of which 56 contributed to the meta‐analyses and five were included exclusively in the qualitative synthesis (Figure 1).

**FIGURE 1:**
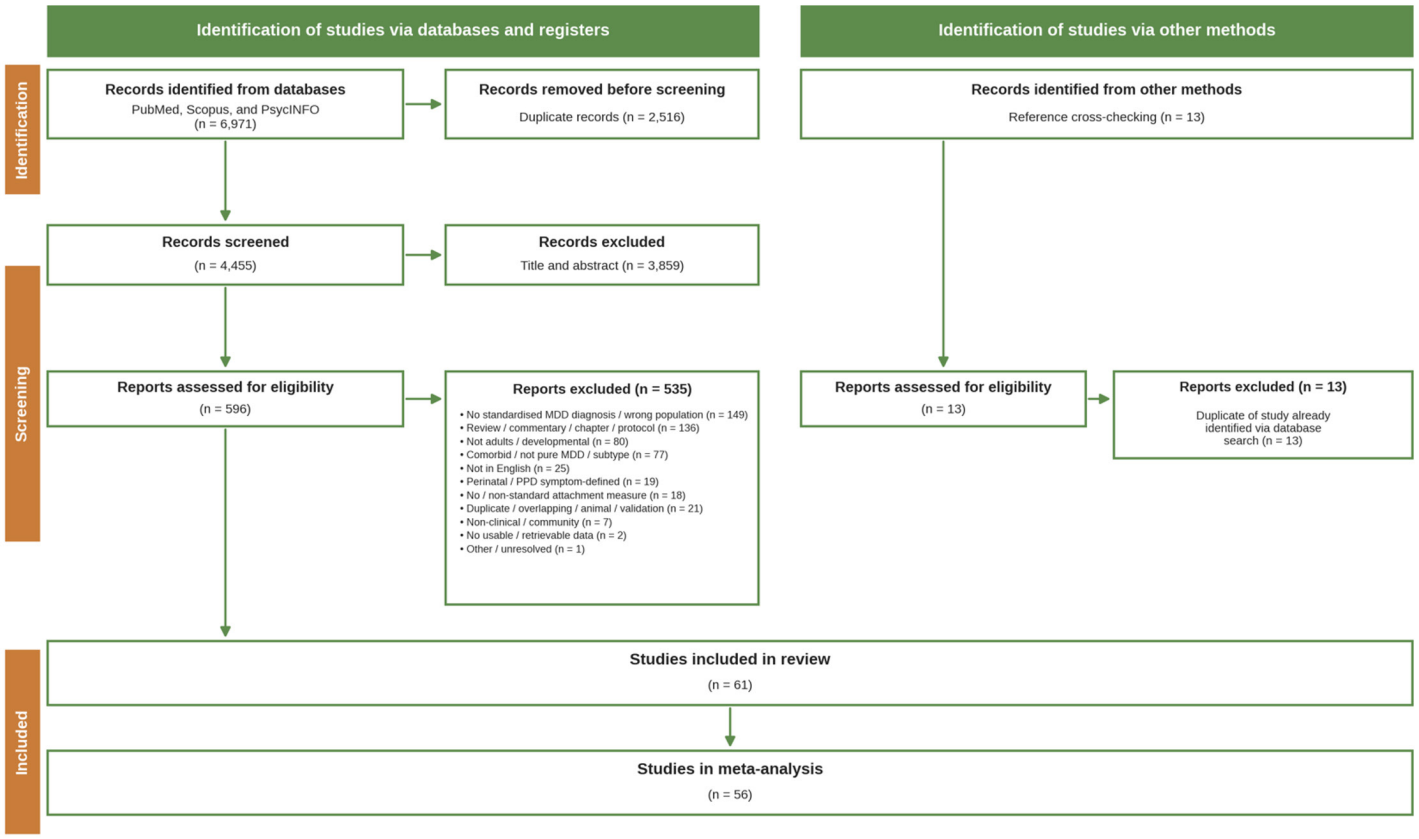
PRISMA 2020 flow diagram for the systematic review and meta‐analysis of attachment patterns in major depressive disorder (MDD). Records were identified through database searches (PubMed, Scopus and APA PsycINFO) and through manual reference‐list (citation) checking. Reference checking identified 13 records, all of which were duplicates of records already retrieved through the database searches, so no additional studies were added by this route. A total of 61 studies met all eligibility criteria and were included in the review, all identified through the database searches, of which 56 contributed extractable effect‐size data to the quantitative meta‐analyses. Five studies (Melnikov et al. 2021; Smith et al. 2012; Watson et al. 2014; Nowak et al. 2026; Cho et al. 2025) met eligibility but could not be entered into the pooled analyses because they did not report extractable effect‐size data (group means and standard deviations, or attachment‐category counts) for the outcomes of interest; they are retained in the PRISMA flow and qualitative synthesis for transparency but do not contribute to the forest plots or pooled estimates.

### 2.4 Data Extraction

Source characteristics that were extracted include: author, publication year and country of data collection. Sample‐level characteristics extracted include participant mean age, gender, race, relationship status and education. Study‐level data extracted include sample size, diagnostic criteria, attachment scale used (Table S1), whether the study investigates PPD and relevant outcome data (means, standard deviations, proportions). For longitudinal studies, we extracted data only from the first assessment because this wave typically had the largest sample size. Where essential data for effect size calculation (e.g., cell counts, means/standard deviations [SDs], sample sizes) were missing, corresponding authors were contacted twice over 4 weeks. If data remained unavailable, the study was excluded from the relevant analysis.

The Newcastle‐Ottawa Scale for case–control studies (Wells et al. 2011) was used to assess the quality of studies with a healthy control (HC) group. For studies without a HC group, we applied the cross‐sectional adaptation of the Newcastle‐Ottawa Scale (Herzog et al. 2013; Modesti et al. 2016), which omits control‐related items. Quality was assessed across three categories: sample selection, comparability (whether confounding factors are controlled for) and outcome (validity of outcome (attachment style) measure and statistical analysis), where a maximum of 10 points can be awarded in total. For both versions of the scale, the methodological quality is considered low if the score is within the range of 0–4, moderate within the range of 5–6 and good for scores ≥ 7 (Abesig et al. 2020; Herzog et al. 2013).

### 2.5 Statistical Analysis

We synthesised categorical and continuous outcomes in separate analyses. Categorical data included the number of participants classified into the four attachment styles: secure, anxious‐preoccupied, dismissing‐avoidant and fearful (Bartholomew and Horowitz 1991) (Table S2). The counts were synthesised using odds ratios comparing MDD/PPD to HC groups. Continuous data consisted of mean and standard deviation (SD) scores on validated attachment scales that mostly follow the two‐factor model of attachment anxiety and attachment avoidance (e.g., Experiences in Close Relationships (ECR); Brennan et al. 1998). For studies implementing multidimensional measures, we calculated composite scores from these scales to reflect the two higher‐order dimensions of attachment anxiety and attachment avoidance (Supporting Information, Merging Dimensions). For studies reporting mean scores for the four attachment styles (i.e., secure, anxious‐preoccupied, dismissing‐avoidant, fearful), fearful and secure constructs were retained under their original labels as secondary outcome (Herstell et al. 2021).

Random‐effects meta‐analysis was employed for both types of measures. Effect sizes were calculated as log odds ratios (OR) for categorical measures and standardised mean differences (SMD) for continuous measures between MDD/PPD and HC groups. As exploratory analyses, we conducted meta‐analyses separately for each scale having at least three contributing studies. We also conducted planned subgroup analyses comparing PDD and MDD studies.

Random‐effects meta‐analyses were fitted using restricted maximum likelihood (REML) estimation of the between‐study variance (*τ* ^2^) to account for expected heterogeneity in study populations, measures and designs. Categorical outcomes were expressed as ORs with 95% confidence intervals (CIs), transformed to the log OR scale for analysis and back‐transformed for interpretation; the Haldane–Anscombe correction (+0.5 added to each cell) was applied where studies contained zero cells. Continuous outcomes were expressed as Hedges' g SMDs as a correction for small‐sample bias. Pooled estimates and their CIs were computed under the normal approximation (z‐distribution). As a sensitivity analysis, the Hartung‐Knapp‐Sidik‐Jonkman adjustment was applied to obtain t‐distributed confidence intervals for the pooled outcomes (Table S3). All tests were two‐tailed with *α* = 0.05. Heterogeneity was quantified using the *I* ^2^ statistic, *τ* ^2^ and Cochran's Q test, with *I* ^2^ values of approximately 25%, 50% and 75% interpreted as low, moderate and high heterogeneity, respectively (Higgins et al. 2003). Where a single study was identified as an outlier, the pooled effect was re‐estimated with that study removed and leave‐one‐out analyses were used to examine the influence of individual studies. Publication bias was assessed through funnel plot inspection and Egger's regression test for outcomes with more than 10 contributing studies, with trim‐and‐fill applied where significant asymmetry was detected. Statistical analyses were conducted in R (version 4.5.1) using the metafor (Viechtbauer 2010), meta (Schwarzer 2007) and clubSandwich (Pustejovsky 2022) packages.

As a secondary analysis, we estimated the prevalence of each attachment style using random‐effects single‐arm meta‐analyses of proportions. All MDD/PPD samples reporting mutually exclusive categorical attachment classifications were eligible, irrespective of whether a healthy‐control group was included. Proportions were pooled on the logit scale using a random‐intercept logistic model (binomial–normal generalised linear mixed model), which avoids the variance instability of untransformed proportions near the boundaries and requires no continuity correction for zero cells. Pooled estimates were back‐transformed to the proportion scale for presentation. Between‐study heterogeneity was quantified using *τ* ^2^, Cochran's Q and *I* ^2^ and 95% prediction intervals were derived to describe the dispersion of true prevalences across settings. Inverse‐variance random‐effects models with REML estimation on the logit scale were fitted as a sensitivity analysis and planned subgroup analyses compared MDD with PPD samples. As Bifulco et al. (2019) applied non‐mutually‐exclusive criteria to the insecure subtypes, they contributed only to the secure versus insecure classification, as in the primary analysis.

Sensitivity analyses were conducted for these single‐arm estimates. For categorical data, the pooled prevalence of secure attachment was compared with a series of general‐population reference values, expressing each comparison as an odds ratio. We used two published estimates: 55% secure (Shaver and Clark 1994) and 59% secure in a nationally representative sample of American adults (Mickelson et al. 1997) and varied the reference from 40%–65% to establish the value below which the comparison would cease to be significant (Table S4b). The prevalence analyses were also repeated using only the patient arms of the case–control studies to test whether the pooled estimates depended on the inclusion of depression‐only samples (Table S4a). For continuous data, scores from the studies without a HC group were rescaled to a 0–100 Percentage of Maximum Possible metric and pooled by random‐effects meta‐analysis, with no healthy‐control value assumed and compared with the correspondingly rescaled patient arms of the double‐arm studies (Tables S5, S6).

## 3 Results

Sixty‐one studies met the inclusion criteria for the systematic review (Table S7). The total patient sample was *n* = 5450 (MDD *n* = 5105; PPD *n* = 345) and healthy controls was *n* = 2344 (MDD‐HC *n* = 1999; PPD‐HC *n* = 345). Of these, 33 studies included both MDD/PPD and HC groups and were eligible for the primary case–control meta‐analyses (Table 1, Table S7), whereas 28 were depression‐only studies without an HC group. The primary meta‐analyses were restricted to studies providing extractable case–control data, with the number of studies contributing varying according to attachment outcome. Five studies (Smith et al. 2012; Watson et al. 2014; Melnikov et al. 2021; Cho et al. 2025; Nowak et al. 2026) met the inclusion criteria, but did not provide extractable data for meta‐analysis because attachment was reported as a predictor, correlate or model output rather than as an extractable between‐group outcome (Supporting Information, Qualitative Synthesis).

**TABLE 1:** Study characteristics.

First author	Year	Country	Patients (*n*)	Healthy controls (*n*)	Patients (M/F)	Healthy controls (M/F)	Patient age	Healthy control Age	Diagnostic criteria	Assessment interview	MDD/PPD	Attachment scale
A‐Tjak	2020	Netherlands	82	0	NR	NA	NR	NR	DSM‐IV	SCID	MDD	ECR
Aceti^a^	2012	Netherlands	22	22	0/22	0/22	37	34	DSM‐IV	SCID	PPD	ECR
Bartolomei	2020	Switzerland	234	0	87/147	NA	41.3	NA	DSM‐IV	SCID	MDD	AAS
Bauriedl‐Schmidt	2017	Germany	23	29	10/13	15/14	43.1	46.3	DSM‐IV	SCID	MDD	AAP
Bernecker	2016	Canada	69	0	17/52	NA	38.2	NA	DSM‐IV	SCID	MDD	ECR‐R, RSQ
Bifulco^b^	2002	UK	89	0	0/89	NA	36.1	NA	DSM‐III‐R	PSE	MDD	ASI
Bifulco	2019	UK	75	127	23/52	57/70	56.94	58.69	DSM‐5	Clinical interview	MDD	VASQ
Bracht^a^	2022	Switzerland	48	18	26/22	10/8	43.2	41.6	DSM‐IV	MINI	MDD	AAS‐R
Cacciola^b^	2020	Sweden	37	0	0/37	NA	32.5	NA	DSM‐IV	PRIME‐MD	PPD	ASQ
Çelikbaş^a^	2022	Turkey	124	144	47/77	68/76	36.7	NR	DSM‐5	Clinical interview	MDD	RSQ
Cheng	2025	China	62	0	31/31	NA	45	NA	ICD‐10	Clinical interview	MDD	ECR‐RS
Cho	2025	South Korea	582	0	168/414	NA	44.7	NA	DSM‐5	Clinical interview	MDD	ECR
Conradi^b^	2009	Netherlands	213	0	73/140	NA	42.5	NA	DSM‐IV	CIDI	MDD	ECR
Costa^a^	2009	Italy	93	192	30/63	59/133	44.3	42.2	DSM‐IV	SCID	MDD	ASQ
Craba^a^	2023	Italy	51	60	15/36	24/36	52.7	52.1	DSM‐V‐TR	Clinical interview	MDD	ECR
Dagnino	2017	Chile	70	0	12/58	NA	42.5	NA	ICD‐10	Clinical interview	MDD	ECR
DiFilippo	2002	USA	84	0	28/56	NA	39.4	NA	DSM‐IV	SCID‐I	MDD	ECR
Demıray^a^	2025	Turkey	64	64	16/48	20/44	32.4	33.1	DSM‐5	Clinical interview	MDD	AASS
Fuhr	2017	Germany	61	60	NR	NR	NR	NR	DSM‐IV	SCID	MDD	RSQ
Gilbert	2009	UK	62	0	26/36	NA	44.3	NA	ICD	Clinical interview	MDD	ECR
Golshani^b^	2021	Iran	300	0	176/124	NA	31.3	NA	DSM‐V	Clinical interview	MDD	AAS
Grunebaum	2010	USA	136	0	54/82	NA	37	NA	DSM‐IV	SCID	MDD	AAS
Huang^b^	2019	Taiwan	17	0	7/10	NA	54.7	NA	DSM‐IV‐TR	MINI	MDD	RQ
Huh	2020	Korea	294	0	134/160	NA	35.5	NA	DSM‐IV	MINI	MDD	ECR
Ihme	2022	France	54	0	20/34	NA	44.4	NA	DSM‐IV	Clinical interview	MDD	ECR‐R
Ikeda^a^	2014	Japan	16	60	0/16	0/60	34.2	33.2	DSM‐IV	MINI	PPD	ASI
Kani	2023	Turkey	38	32	5/33	10/22	37.9	37.7	DSM‐5	SCID	MDD	AAS
Karabekiroğlu^a^	2011	Turkey	32	174	10/22	93/81	30.6	NR	DSM‐IV	SCID	MDD	AAS
Koelkebeck^a^	2017	Germany	38	40	21/17	23/17	40.3	39.7	DSM‐IV	SCID	MDD	AAS
König	2026	Germany	76	76	22/54	22/54	27.58	27.63	DSM‐5	SCID‐5‐CV	MDD	RSQ
Kwon^a^	2017	South Korea	52	31	17/35	9/22	40.4	38.7	DSM‐IV	SCID	MDD	ASI
Levitan^b^	2009	Canada	199	0	74/125	NA	39.1	NA	DSM‐IV	SCID	MDD	AAS
Marazziti^a^	2007	Italy	22	126	10/12	58/68	40.1	33.2	DSM‐IV	SCID	MDD	ECR
Martínez	2023	Chile	178	0	66/112	NA	36.9	NA	ICD‐10	Clinical interview	MDD	ECR
Martucci^a^	2021	Italy	46	28	0/46	0/28	36	NR	DSM‐V	Clinical interview	PPD	ECR‐R
McBride^a^	2006	Canada	75	151	23/52	75/76	NR	NR	DSM‐IV	SCID	MDD	RSQ
McMahon	2005	Australia	62	38	0/62	0/38	31.4	31.4	DSM‐IV	CIDI	PPD	ASQ
Melnikov	2021	Russia	21	21	6/15	6/15	34.3	33.8	ICD‐10	Clinical interview	MDD	ECR
Murray^a^	2006	UK	51	40	0/51	0/40	NR	NR	DSM‐IV	SADS, SCID	PPD	AAI
Niolu^a^	2016	Italy	49	52	0/49	0/52	60.2	58	DSM‐IV/ICD‐10	MINI Plus	MDD	RQ
Nonnenmacher	2016	Germany	34	59	0/34	0/59	NR	NR	DSM‐IV	SCID	PPD	ASI
Nowak	2026	Germany	74	0	24/50	NA	36.3	NA	DSM‐IV	SCID‐I/II	MDD	ECR
Oon‐arom	2019	Thailand	119	0	69/50	NA	69.57	NA	DSM‐IV	MINI	MDD	ECR‐R
Özaslan^a^	2025	Turkey	118	92	34/84	32/60	42	40.9	DSM‐5	Clinical interview	MDD	ECR‐II
Özer^a^	2015	Turkey	62	60	7/55	6/54	34.7	NR	DSM‐IV‐TR	SCID	MDD	ECR
Paradiso^a^	2012	USA	73	47	29/44	19/28	60.2	70.2	DSM‐IV‐TR	SCID	MDD	ECR
Picardi^a^	2019	Italy	21	21	11/10	11/10	53.2	53.3	DSM‐IV‐TR	SCID	MDD	ECR
Reinecke	2001	USA	54	0	23/31	NA	38	0	DSM‐IV	SCID	MDD	RAAS
Reiner^a^	2016	Germany	43	42	0/43	0/42	NR	NR	DSM‐IV	SCID	MDD	AAI
Reis^a^	2004	Australia	71	245	28/43	59/186	45.03	21.38	DSM‐IV	SCID	MDD	RQ
Santona^a^	2015	Italy	30	30	0/30	0/30	31.5	NR	DSM‐5	SCID	PPD	AAI
Smith	2012	USA	8	0	0/8	NA	40.38	NA	DSM‐IV	SCID	MDD	ECR
Spence^b^	2020	UK	36	0	0/36	NA	61.4	NA	DSM‐IV	SCID	MDD	ASI
Strauss	2006	Germany	109	0	33/77^c^	NA	34.5	NA	ICD‐10	Clinical interview	MDD	AAPR
Strecker^a^	2025	Germany	24	35	6/18	5/30	40.1	39.6	DSM‐IV	Mini‐DIPS	MDD	RSQ
Troxel^b^	2007	USA	107	0	0/107	NA	38.4	NA	DSM‐IV	SADS or SCID	MDD	RQ
Watson^b^	2014	Canada	55	0	18/37	NA	37.6	NA	DSM‐IV	Clinical interview	MDD	ASQ
Whiffen^a^	2001	Canada	52	60	0/52	0/60	37.4	36.5	DSM‐III‐R	SADS	MDD	RQ
Wilkinson^a^	2010	Australia	47	68	0/47	0/68	32.3	31.2	DSM‐IV	MCMI‐III	PPD	RQ
Yotsidi	2024	Greece	273	0	93/180	NA	44.1	NA	DSM‐5	MINI	MDD	ECR
Zhang	2023	China	139	0	63/76	NA	31.4	NA	DSM‐V	Clinical interview	MDD	RAAS

*Note:* Mean age is presented for patients and controls.

Abbreviations: AAI, Adult Attachment Interview; AAS, Adult Attachment ScaleAAS‐R, Adult Attachment Scale–Revised; AASS, Adult Attachment Style Scale; ASI, Attachment Style Interview; ASQ, Attachment Style Questionnaire; CIDI, Composite International Diagnostic Interview; DSM, Diagnostic and Statistical Manual of Mental Disorders; ECR, Experiences in Close Relationships; ECR‐II, Experiences in Close Relationships Inventory–II; ECR‐R, Experiences in Close Relationships–Revised; ICD, International Classification of Diseases; MCMI‐III, Millon Clinical Multiaxial Inventory 3rd Edition; MDD, major depressive disorder; M/F, male/female; MINI, Mini‐International Neuropsychiatric Interview; Mini‐DIPS, Mini‐DIPS Diagnostic Short‐Interview for Mental Disorders; MINI Plus, Mini‐International Neuropsychiatric Interview Plus; NA, not applicable; NR, not reported; PPD, postpartum depression; PRIME‐MD, Primary Care Evaluation of Mental Disorders; PSE, Present State Examination; RAAS, Revised Adult Attachment Scale; RQ, Relationship Questionnaire; RSQ, Relationship Scales Questionnaire; SADS, Schedule for Affective Disorders and Schizophrenia; SCID, Structured Clinical Interview for DSM; VASQ, Vulnerable Attachment Style Questionnaire.

^a^ Studies included in the primary meta‐analyses.

^b^ Studies included in the sensitivity analyses: Reiner et al. (2016) included one dysthymic patient in their sample.

^c^ Strauss et al. (2006) gender distribution is estimated from the full sample with multiple diagnoses (*n* = 617) which was 70% women (mean age 34.5 years and age range from 16–65 years). Only McBride et al. (2006), which includes a healthy control group, contributed to the meta‐analyses. Oon‐arom et al. (2019) consisted of major depressive disorder and dysthymia which were combined and not separately reported. Cacciola and Psouni (2020) used a structured diagnostic instrument, the Primary Care Evaluation of Mental Disorders (PRIME‐MD), a DSM‐IV‐based clinical instrument, though DSM‐IV was not directly stated in the publication. Wilkinson and Mulcahy (2010) used the MCMI‐III, a DSM‐IV‐aligned self‐report inventory assessing clinical syndromes and personality disorders. Melnikov et al. (2021) used a Russian version of the ECR. Reis and Grenyer (2004) used a non‐clinical university student sample as the comparison group rather than a screened healthy control group.

Race and ethnicity were reported by a small number of studies, primarily those conducted in USA (e.g., Reinecke and Rogers (2001): 61% Caucasian and 35% African American; Troxel et al. (2007): approximately 88% non‐Hispanic White). Most studies did not report participant race or ethnicity (Table S8).

Attachment styles had been measured using the following scales: the Adult Attachment Interview (George et al. 1985; Main et al. 2002), Adult Attachment Projective (AAP; George and West 2001), Adult Attachment Prototype Rating (AAPR; Strauss et al. 1999), Adult Attachment Scale (AAS; Collins and Read 1990), Adult Attachment Scale‐Revised (AAS‐R; Schmidt et al. 2016), Revised Adult Attachment Scale (RAAS; Collins 1996), Adult Attachment Style Scale (AASS; Mikulincer et al. 1990), Attachment Style Interview (ASI; Bifulco et al. 2002), Attachment Style Questionnaire (ASQ; Feeney et al. 1994), Experiences in Close Relationships (ECR; Brennan et al. 1998), Experiences in Close Relationships‐Revised (ECR‐R; Fraley et al. 2000), Experiences in Close Relationships Inventory‐II (ECR‐II; Turkish adaptation of the ECR‐R, Selçuk et al. 2005), Experiences in Close Relationships‐Relationship Structures (ECR‐RS; Fraley et al. 2011), Relationship Questionnaire (RQ; Bartholomew and Horowitz 1991), Relationship Scales Questionnaire (RSQ; Griffin and Bartholomew 1994) and Vulnerable Attachment Style Questionnaire (VASQ; Bifulco et al. 2003).

Quality was assessed as good (12 studies), moderate (13 studies) and poor (eight studies) in the studies which included both MDD/PPD and HC. Quality was assessed as good (13 studies), moderate (13 studies) and poor (two studies) in the depression‐only studies without a HC group (Table S9). Funnel plots for publication bias showed no significant asymmetry for the anxious‐continuous (*t* = 2.00, df = 13, *p* = 0.067), avoidant‐continuous (*t* = 0.52, df = 13, *p* = 0.610), anxious‐categorical (*t* = 1.43, df = 14, *p* = 0.174) and avoidant‐categorical (*t* = 0.48, df = 14, *p* = 0.637) models, whereas significant asymmetry was detected for the secure‐categorical (*t* = −3.04, df = 19, *p* = 0.007) and fearful‐categorical (*t* = 2.28, df = 10, *p* = 0.046) models (Figure S1). Subsequent trim‐and‐fill analyses indicated that both associations were robust: for secure attachment, four potentially missing studies were imputed on the right side of the funnel, yielding an adjusted pooled odds ratio of 0.23 (95% CI [0.17, 0.30], *p* < 0.001); for fearful attachment, two potentially missing studies were imputed on the left, yielding an adjusted pooled odds ratio of 2.68 (95% CI [1.71, 4.22], *p* < 0.001), indicating that both associations remained significant despite evidence of asymmetry.

### 3.1 Meta‐Analysis

The meta‐analysis consisted of 33 studies that reported both MDD/PPD and HC groups (Table 1). Detailed demographics of the meta‐analysis can be found in Table 2. Of all studies, three contributed to some outcomes due to missing required subscales (McMahon et al. 2005), reporting only factor structures (Fuhr et al. 2017), applying non‐mutually‐exclusive categorical criteria (Bifulco et al. 2019). One further study reported no extractable attachment data (Melnikov et al. 2021). The remaining 28 studies did not include a HC group; of these, eight reported mutually exclusive categorical attachment classifications and were eligible for the secondary single‐arm prevalence analyses, 16 reported dimensional scores only, which cannot be pooled without a HC comparator (Table S7) and four reported no extractable attachment data (Cho et al. 2025; Nowak et al. 2026; Smith et al. 2012; Watson et al. 2014) (Table S7).

**TABLE 2:** Demographics of the meta‐analysis.

Characteristic	Full sample	MDD	PPD
Number of studies	61	52	9
Depression group			
Participants, *n*	5450	5105	345
Mean age, years	40.7	41.1	33.2
Female, % (*n/N*)	68.1 (3616/5308)	65.9 (3271/4963)	100.0 (345/345)
Healthy control group			
Participants, *n*	2344	1999	345
Mean age, years	38.5	39.4	32.2
Female, % (*n/N*)	70.2 (1603/2284)	64.9 (1258/1939)	100.0 (345/345)

*Note:* Number of participants is presented; mean age is presented in years. Weighted means are computed using study‐level sample sizes as weights. The Female row shows the number of female participants over the number of participants for whom sex was reported (n/N); this denominator is smaller than the total participant count because studies that did not report a sex breakdown are excluded from the Female *N* while still contributing to the total. Mean age was available for 55 of the 61 depression‐group samples and for 23 of the 33 studies with a healthy‐control arm; sex distribution was available for 59 of the 61 depression‐group samples (all except A‐Tjak and Fuhr) and for 32 of the 33 control arms (all except Fuhr). Six depression‐group studies did not report mean age (A‐Tjak, Fuhr, McBride, Murray, Nonnenmacher, Reiner). Healthy‐control rows reflect only the case–control subset: 28 of the 61 included studies were single‐arm depression‐only designs without a healthy‐control comparator. Participant counts are the total samples recruited by each included study (as listed in Table 1) and exceed the effective sample sizes entered into the pooled meta‐analyses (patients *n* ≈ 5336; healthy controls *n* ≈ 2276), because several studies contributed only a subsample to the quantitative synthesis (e.g., comorbidity‐free or diagnostically defined subgroups) and five qualitative‐only studies provided no extractable effect‐size data. PPD studies were 100% female by sampling‐frame definition. MDD = major depressive disorder; PPD = peripartum depression. For Bifulco et al. (2002), only the depressed subsample (*n* = 89) was entered; the non‐depressed women from that study were not used as healthy controls, because they were drawn from the same high‐risk community sample rather than a separate healthy comparison group.

#### 3.1.1 Continuous Outcomes

Attachment style scores in both MDD/PPD and HC were measured in 16 studies (MDD/PPD *n* = 959, HC *n* = 1435), using the following scales: AAS/AAS‐R (*n* = 3), ASQ (*n* = 2), ECR/ECR‐R (*n* = 4), RQ (*n* = 3) and RSQ (*n* = 4).

In the primary analysis, secure attachment scores were significantly reduced in MDD/PPD compared to HC (*k* = 7, Hedges' g = −0.46, 95% CI: [−0.77; −0.16], *z* = −2.96, *p* = 0.003), with substantial heterogeneity (*I* ^2^ = 86.0%) (Figure 2a).

**FIGURE 2:**
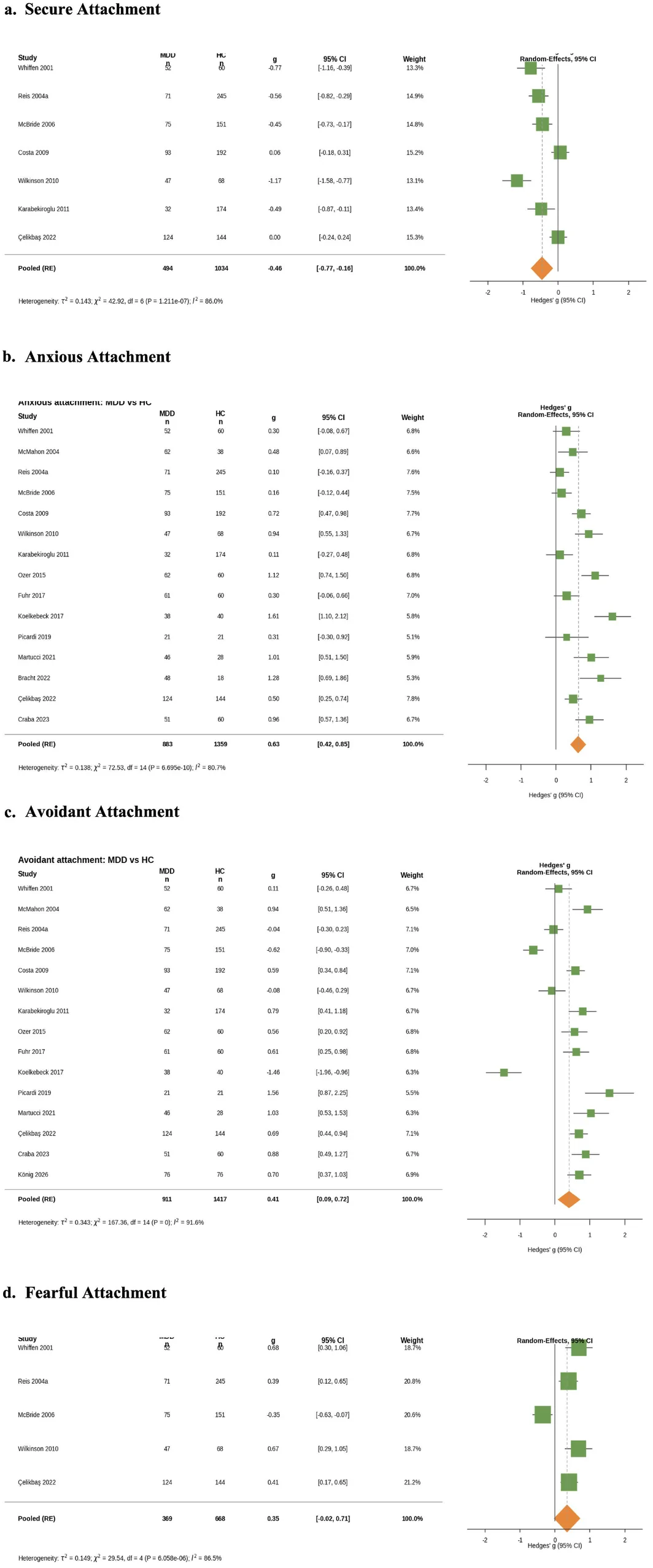
Forest plots of continuous attachment outcomes in major depressive disorder (MDD) versus healthy controls (HC). Each panel shows a random‐effects meta‐analysis of standardised mean differences (Hedges' g) for one attachment dimension: (a) secure (k = 7; MDD *n* = 494, HC *n* = 1034); (b) anxious (*k* = 15; MDD *n* = 883, HC *n* = 1359); (c) avoidant (*k* = 15; MDD *n* = 911, HC *n* = 1417); (d) fearful (k = 5; MDD *n* = 369, HC *n* = 668). For each study, the green square represents the estimated effect size (Hedges' g), with size proportional to the study's weight in the pooled analysis and the horizontal line indicates the 95% confidence interval. The orange diamond at the bottom of each plot represents the pooled estimate and its 95% confidence interval. Positive values of g indicate higher scores in the MDD group than in HC; negative values indicate lower scores in MDD. Effect sizes were calculated as Hedges' g with the small‐sample bias correction. Pooled estimates were obtained using a random‐effects model with between‐study variance (*τ* ^2^) estimated by restricted maximum likelihood (REML); confidence intervals around the pooled estimate were derived under the normal approximation (Wald z‐distribution); Hartung–Knapp‐adjusted intervals are provided as a sensitivity analysis in Table S3. Heterogeneity is summarised below each plot using *τ* ^2^, Cochran's Q (with degrees of freedom and *p*‐value) and the *I* ^2^ statistic. The vertical solid line marks the null effect (*g* = 0); the vertical dotted line marks the pooled effect estimate. MDD = number of major depressive disorder participants in each study; HC = number of healthy control participants; *g* = Hedges' g (standardised mean difference); 95% CI = 95% confidence interval; weight = percentage contribution to the random‐effects pooled estimate.

Anxious attachment was significantly greater in MDD/PPD compared to HC (*k* = 15, Hedges' *g* = 0.63, 95% CI: [0.42; 0.85], *z* = 5.80, *p* < 0.001) with high heterogeneity (*I* ^2^ = 80.7%) (Figure 2b). Avoidant attachment was significantly greater in MDD/PPD compared to HC (*k* = 15, Hedges' g = 0.41, 95% CI: [0.09; 0.72], *z* = 2.54, *p* = 0.011) with high heterogeneity (*I* ^2^ = 91.6%) (Figure 2c). Fearful attachment showed no significant difference between MDD/PPD and HC (k = 5, Hedges' g = 0.35, 95% CI: [−0.02; 0.71], *z* = 1.87, *p* = 0.062) with high heterogeneity (*I* ^2^ = 86.5%) (Figure 2d); however, fearful attachment was significantly greater in MDD/PPD relative to HC when one outlier study was removed, which had reported greater fearful attachment in HC (McBride et al. 2006) (*k* = 4, Hedges' g = 0.48, 95% CI: [0.34; 0.63], *z* = 6.36, *p* < 0.001) with no heterogeneity (*I* ^2^ = 0%). One study (Reis and Grenyer 2004) had a non‐clinical university student sample as its comparison group rather than a screened HC group; in a leave‐one‐out sensitivity analysis, removing this study left the pooled continuous SMD essentially unchanged (all|Δg| ≤ 0.05), indicating that the continuous findings do not depend on this comparator.

Exploratory subgroup analyses based on different attachment measures were further conducted for scales with at least three contributing studies (ECR‐family and RQ) (Table S10a).

#### 3.1.2 Categorical Outcomes

The primary analysis included 21 studies for secure attachment (MDD/PPD *n* = 997, HC *n* = 1214), 16 studies each for anxious and avoidant (MDD/PPD *n* = 794, HC *n* = 978) and 12 studies for fearful (MDD/PPD *n* = 606, HC *n* = 802) attachment. Scales used were ECR (*k* = 6), ASI (*k* = 3), AAI (*k* = 3), RQ (*k* = 3), RSQ (*k* = 2), AASS (*k* = 1) and ECR‐R (*k* = 1).

Secure attachment was significantly reduced in MDD/PPD (25.4%) compared to HC (63.4%, *k* = 21) (Figure 3). Pooled across all double‐arm studies, secure attachment was significantly reduced in MDD/PPD (OR = 0.20, 95% CI: [0.15; 0.26], *z* = −11.35, *p* < 0.001), with moderate heterogeneity (*I* ^2^ = 43.3%) (Figure 4a). Sensitivity analysis included an additional 8 depression‐only studies without a healthy‐control group (*n* = 908; combined *k* = 29), confirming the significant reduction in secure attachment in MDD/PPD. Relative to general‐population reference values for secure attachment of 50%–60%, the pooled odds of secure attachment in MDD/PPD were significantly reduced (OR = 0.33, 95% CI: [0.25; 0.42] against a reference of 50% and OR = 0.23, 95% CI: [0.18; 0.29] against 59%) and the reduction would persist against any reference value above 29.4% (Table S4b).

**FIGURE 3:**
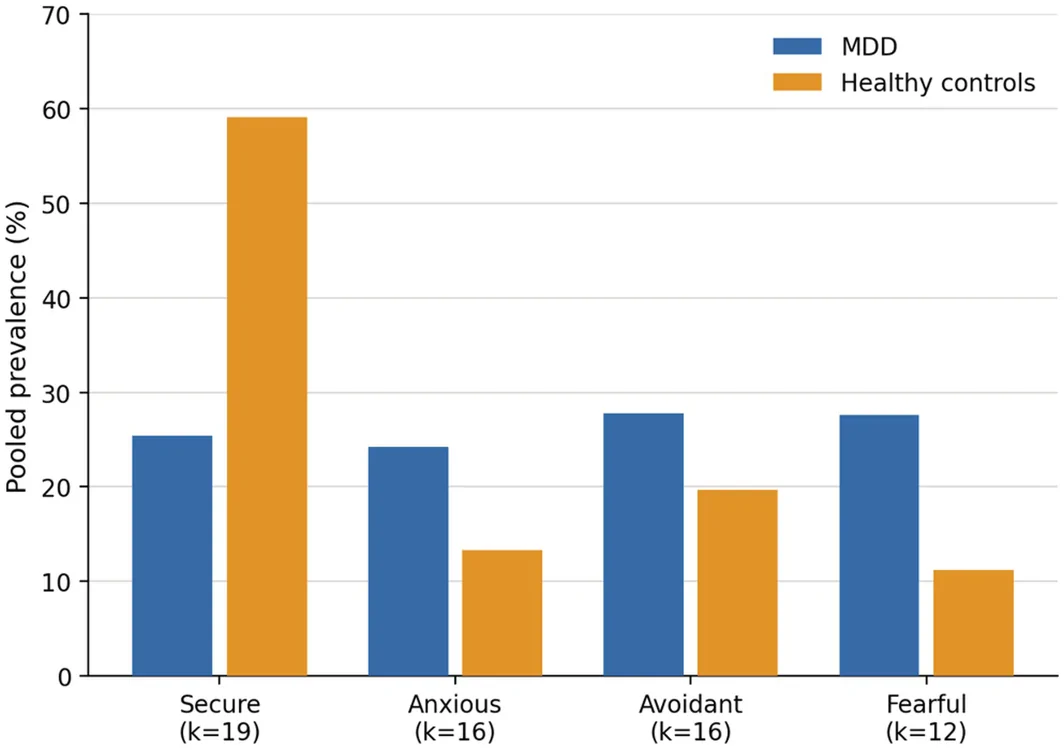
Pooled prevalence rates of attachment styles in MDD and healthy controls. Bars show the percentage of participants classified within each attachment style among studies reporting prevalence data for both major depressive disorder (MDD) and healthy control (HC) groups. Blue bars represent MDD participants and amber bars represent healthy controls. Secure attachment was less prevalent in MDD than in HC groups (25.4% vs. 59.1%; OR = 0.20, 95% CI 0.15–0.26, *p* < 0.001; *k* = 21). Anxious/preoccupied attachment was more prevalent in MDD than in HC groups (24.2% vs. 13.3%; OR = 2.46, 95% CI 1.66–3.64, *p* < 0.001; *k* = 16), as was fearful attachment (27.6% vs. 11.2%; OR = 3.00, 95% CI 1.97–4.54, *p* < 0.001; *k* = 12). Avoidant/dismissive attachment was also more prevalent in MDD than in HC groups, with a small but significant effect that became borderline under the Hartung–Knapp adjustment (27.8% vs. 19.7%; OR = 1.42, 95% CI 1.04–1.95, *p* = 0.027; *k* = 16). MDD = major depressive disorder; HC = healthy controls; OR = odds ratio; CI = confidence interval; *k* = number of contributing studies. Bars show the pooled prevalence of each attachment style estimated by random‐effects meta‐analysis of proportions (random‐intercept logistic model, logit scale), with error bars showing 95% confidence intervals. The same estimator and the same study set are used for every style and for both groups: secure *k* = 21, anxious *k* = 16, avoidant *k* = 16, fearful *k* = 12, corresponding to the studies in Figure 4. MDD = major depressive disorder; PPD = postpartum depression.

**FIGURE 4:**
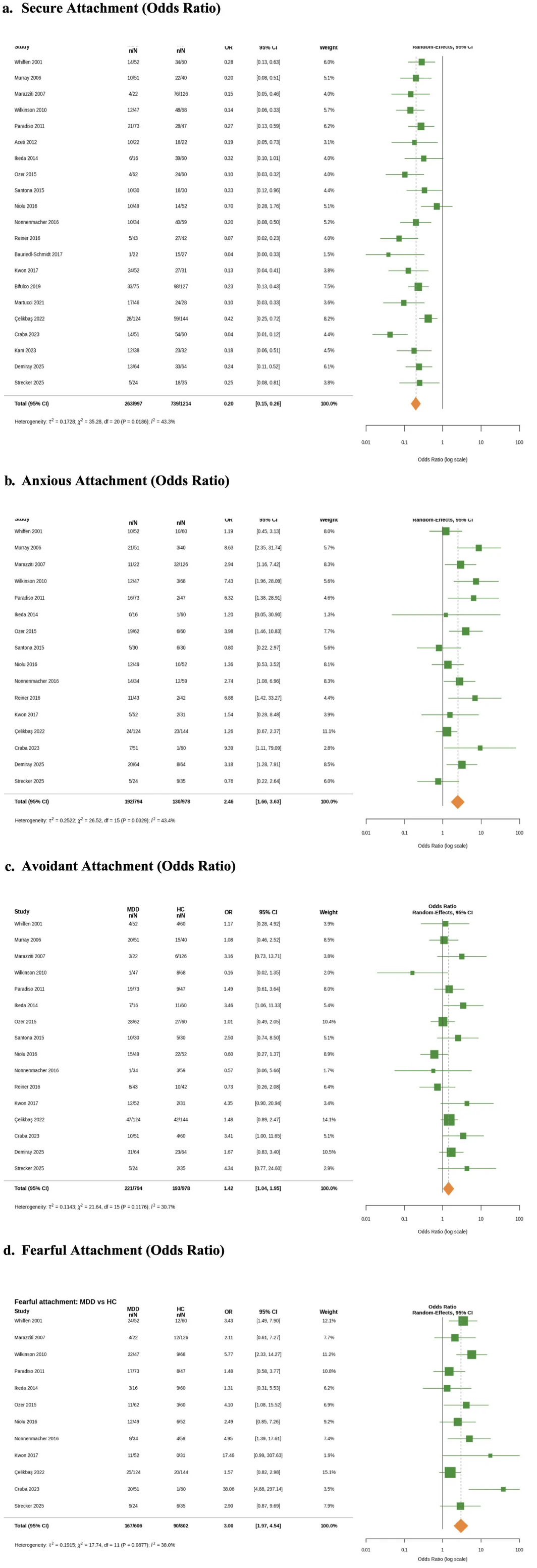
Forest plots of categorical outcomes for all attachment styles. (a) Forest plot for secure attachment (odds ratio) pooled odds ratio comparing the prevalence of secure attachment in patients with MDD versus healthy controls (HC), across *k* = 21 studies. Secure attachment was significantly less common in MDD (OR = 0.20, 95% CI 0.15–0.26, z = −11.35, *p* < 0.001), with moderate heterogeneity (*τ* ^2^ = 0.1728; *χ* ^2^ = 35.28, df = 20, *p* = 0.018; *I* ^2^ = 43.3%). (b) Forest plot for anxious attachment (odds ratio) pooled odds ratio comparing the prevalence of anxious/preoccupied attachment in patients with MDD versus healthy controls, across *k* = 16 studies. Anxious attachment was significantly more common in MDD (OR = 2.46, 95% CI 1.66–3.64, *z* = 4.50, *p* < 0.001), with moderate heterogeneity (*τ* ^2^ = 0.2572; *χ* ^2^ = 26.52, df = 15, *p* = 0.033; *I* ^2^ = 43.4%). (c) Forest plot for avoidant attachment (odds ratio) pooled odds ratio comparing the prevalence of avoidant/dismissive attachment in patients with MDD versus healthy controls, across *k* = 16 studies. Avoidant attachment was significantly more common in MDD (OR = 1.42, 95% CI 1.04–1.95, *z* = 2.21, *p* = 0.027), with low‐to‐moderate heterogeneity (*τ* ^2^ = 0.1143; *χ* ^2^ = 21.64, df = 15, *p* = 0.118; *I* ^2^ = 30.7%). (d) Forest plot for fearful attachment (odds ratio) pooled odds ratio comparing the prevalence of fearful attachment in patients with MDD versus healthy controls, across *k* = 12 studies. Fearful attachment was significantly more common in MDD (OR = 3.00, 95% CI 1.97–4.54, *z* = 5.16, *p* < 0.001), with moderate heterogeneity (*τ* ^2^ = 0.1915; *χ* ^2^ = 17.74, df = 11, *p* = 0.088; *I* ^2^ = 38.0%). General notes: Studies are ordered chronologically. Each row presents one study with the number of participants in each attachment category over the total sample (*n*/*N*) for MDD and healthy controls (HC), the individual study odds ratio with 95% confidence interval and the random‐effects weight. Green squares represent individual study point estimates with sizes proportional to their inverse‐variance weights; horizontal whiskers depict 95% confidence intervals. The orange diamond at the bottom shows the pooled odds ratio with its 95% confidence interval. The solid black vertical line at OR = 1 represents the null effect; the dotted vertical line marks the pooled point estimate. Confidence intervals extending beyond the plotted range are indicated by rightward arrows (e.g., Kwon et al. 2017 and Craba et al. 2023 in panel d). Pooled estimates were obtained using random‐effects meta‐analysis with restricted maximum likelihood (REML) estimation of between‐study variance (*τ* ^2^). Haldane–Anscombe continuity correction (+0.5) was applied to studies with zero cells. Heterogeneity is reported below each plot using *τ* ^2^, Cochran's Q statistic with degrees of freedom and *p*‐value and the *I* ^2^ statistic. Abbreviations: CI = confidence interval; HC = healthy controls; MDD = major depressive disorder; OR = odds ratio.

Anxious attachment was significantly increased in MDD/PPD (23.7%) compared to HC (10.5%, *k* = 16, OR = 2.46, 95% CI: [1.66; 3.64], z = 4.50, *p* < 0.001), with moderate heterogeneity (*I* ^2^ = 43.4%) (Figure 4b). Avoidant attachment was significantly more common in MDD/PPD (23.1%) than in HC (16.3%, *k* = 16, OR = 1.42, 95% CI: [1.04; 1.95], *z* = 2.21, *p* = 0.027), with low‐to‐moderate heterogeneity (*I* ^2^ = 30.7%) (Figure 4c). Fearful attachment was significantly greater in MDD/PPD (27.7%) relative to HC (10.4%, *k* = 12, OR = 3.00, 95% CI: [1.97; 4.54], *z* = 5.16, *p* < 0.001), with moderate heterogeneity (*I* ^2^ = 38.0%) (Figure 4d).

Subgroup analyses by diagnostic context (MDD and PPD) revealed a differentiated pattern. In the MDD subgroup, all three insecure attachment patterns (anxious, avoidant and fearful) remained significantly elevated relative to healthy controls, mirroring the primary analysis. In the PPD subgroup, secure attachment was significantly reduced (*k* = 7, OR = 0.19, 95% CI [0.13, 0.29]); anxious (*k* = 5, OR = 3.16 [1.23, 8.10], *p* = 0.017) and fearful attachment (*k* = 3, OR = 3.83 [1.70, 8.64], *p* = 0.001) were also significantly elevated, whereas avoidant attachment was not (*k* = 5, OR = 1.28 [0.54, 3.07]) (Tables S11–S12; Figures S2–S3).

Current‐episode status (current episode versus a range of symptom severity or remission) did not significantly moderate any categorical or continuous attachment outcome, whether examined as a subgroup or as a meta‐regression moderator (Table S13).

Exploratory subgroup analyses based on attachment scale were conducted for scales with at least three contributing studies (RQ, ECR, AAI) (Table S10b). The three additional studies identified in the updated search used the RSQ (Strecker et al. 2025; combined with Çelikbaş and Yalçınkaya‐Alkar 2022 yielding *k* = 2) and the AASS (Demıray and Bacık Yaman 2025; *k* = 1), which fell below the predetermined minimum of three contributing studies and were thus not analysed as separate scale‐specific subgroups.

#### 3.1.3 Prevalence of Attachment Styles in MDD/PPD

Secondary single‐arm meta‐analyses estimated the prevalence of each attachment style within clinically diagnosed depression, pooling all eligible MDD/PPD samples reporting mutually exclusive categorical classifications, irrespective of whether an HC group was included. Secure attachment was present in 24.1% (95% CI 19.8–28.9; *k* = 29; *I* ^2^ = 78.6%), so that approximately three in four patients were classified as insecurely attached. Among the insecure patterns, fearful attachment was 26.9% (95% CI 21.9–32.5; *k* = 20; *I* ^2^ = 72.2%), avoidant/dismissive attachment 23.7% (95% CI 18.0–30.5; *k* = 25; *I* ^2^ = 84.1%) and anxious/preoccupied attachment 23.0% (95% CI 17.8–29.2; *k* = 25; *I* ^2^ = 91.5%) (Figure S4; Table S14).

Heterogeneity was substantial across all four styles and 95% prediction intervals were correspondingly wide (secure 8.6%–51.5%), indicating that the pooled values summarise the central tendency of the literature rather than an expected value in any individual sample.

In subgroup analyses, secure attachment was significantly more common in PPD (31.9%, *k* = 8) than in MDD samples (21.3%, *k* = 21; Qᵇ = 6.91, *p* = 0.009), whereas the three insecure styles did not differ significantly between the two contexts (all *p* ≥ 0.19; Table S15). Restricting the analysis to the patient arms of the case–control studies changed each pooled estimate by no more than 2.5 percentage points (Table S4a), indicating that the prevalence estimates are not driven by the inclusion of single‐arm samples.

The depression‐only studies reporting dimensional rather than categorical data were examined in a parallel analysis on the POMP metric (Tables S5 and S6). Pooled attachment anxiety was 46.8% (95% CI 42.2–51.4; *k* = 17) of the scale range in the depression‐only samples and 47.0% (95% CI 40.9–53.1; *k* = 15) in the patient arms of the case–control studies (Qᵇ = 0.00, *p* = 0.97). The corresponding values for attachment avoidance were 45.8% (95% CI 40.9–50.8; *k* = 14) and 46.0% (95% CI 38.4–53.6; *k* = 15) (Qᵇ = 0.00, *p* = 0.97). The depression‐only samples were indistinguishable from the samples that contribute to the case–control comparisons and the difference did not exceed 2.5 percentage points of the scale range in any sensitivity analysis. As these were pooled raw scores rather than effect sizes, heterogeneity was necessarily high (*I* ^2^ = 95.9%–97.2%) and the pooled values are descriptive rather than comparable with the SMD reported above.

## 4 Discussion

This meta‐analysis provides convergent evidence that individuals with MDD/PPD show marked reductions in secure attachment alongside elevations in all three insecure patterns. Anxious attachment was elevated on both categorical and continuous measures, fearful attachment showed the largest categorical elevation and avoidant attachment was elevated by a smaller margin and less robustly. The findings bring together categorical and continuous measures across both MDD and postpartum samples in a single synthesis.

The stronger and more consistent elevations in anxious and fearful attachment support previous reviews (e.g., Dagan et al. 2018; Herstell et al. 2021; Roberts et al. 1996; Simonelli et al. 2004; Zheng et al. 2020) and the interpersonal framework of depression in which reassurance‐seeking, negative feedback‐seeking, blame maintenance, interpersonal conflict and rejection and stress generation bring about the onset and maintenance of depression (Coyne 1976; Hames et al. 2013; Joiner and Timmons 2009). Together, they suggest that interpersonal difficulties characteristic of MDD reflect hyperactivating attachment strategies, marked by heightened threat vigilance, proximity‐seeking, rumination and negatively biased self‐processing (Bartholomew and Horowitz 1991; Gillath et al. 2005; Mikulincer et al. 2002). This negative self‐perception is linked to self‐criticism and maladaptive attributional styles, which amplify negative affect and reinforce depressive cognitions, creating reciprocal cycles that maintain or worsen depressive symptoms (Shaver and Mikulincer 2002) and predict future depressive episodes (Adams et al. 2009; Hu et al. 2015; Orth et al. 2008). Insecure attachment in MDD is further associated with maladaptive coping, limited access to or use of social support and heightened vulnerability to depressive episodes (Mikulincer et al. 2002), whereas secure attachment functions as a resilience feature that supports flexible emotion regulation, coherent representations of self and others and effective mobilisation of social support (Hammen et al. 1995; Bender and Ingram 2018).

The categorical analysis revealed a greater elevation in fearful than in anxious attachment. The two patterns diverge in their internal models of others: anxious attachment typically retains a relatively positive model of others, allowing distress to be alleviated through proximity‐seeking and reassurance, whereas fearful attachment is characterised by a negative model of others marked by expectations of rejection and mistrust that undermine the perceived value of seeking support (Bartholomew and Horowitz 1991; Khodarahimi et al. 2016). Lacking this relational buffer, individuals with fearful attachment may manage distress on their own, allowing self‐blame and feelings of defectiveness to become more pervasive. The combination of negative models of both self and others may be particularly depressogenic, whereby fearful attachment is associated with more severe symptom presentations and poorer treatment engagement (Degnan et al. 2022).

The present meta‐analysis found avoidant attachment to be significantly elevated in MDD/PPD relative to healthy controls in both the categorical and continuous analyses, although of a smaller magnitude than the elevations in anxious and fearful attachment. A deactivating attachment strategy may dampen distressing emotions and inhibit encoding of emotionally charged information (Fraley et al. 2000), reducing help‐seeking or the reporting of depressive symptoms (Dozier and Kobak 1992; Roisman et al. 2004), which may be clinically relevant for a subset of patients. The avoidant effect was the least robust of the three insecure patterns, becoming borderline under the Hartung–Knapp adjustment and not reaching significance within the PPD subgroup. Within depression, however, avoidant attachment was as common as the other insecure patterns, so its more modest case–control contrast may reflect its relatively higher frequency among healthy controls. How avoidance is measured also plays a part. It was raised on scales that score avoidance separately from anxiety, but not on measures that ask participants to select one of four descriptions as these classify someone who is both anxious and avoidant as fearful rather than dismissing; as a result avoidance is recorded less often and scale family was the only moderator to reach significance in the meta‐regressions.

The single‐arm prevalence analyses complement the case–control comparisons by describing the attachment distribution within MDD/PPD and they incorporate the depression‐only studies that could not contribute to a healthy control contrast. Approximately three in four patients with MDD or PPD were classified as having insecure attachment and the three insecure patterns were of broadly comparable prevalence. Insecure attachment in MDD/PPD is thus common, but no single insecure pattern predominates. Attachment style cannot be inferred from the diagnosis and requires assessment in the individual patient.

The MDD and PPD subgroups showed broadly similar attachment profiles, with two exceptions: secure attachment was more prevalent in PPD than in MDD samples and avoidant attachment was significantly elevated relative to healthy controls in MDD but not in PPD. The convergence in secure, anxious and fearful attachment is notable because depressive symptoms arise in these two contexts in different ways, with PPD following a distinct biological event, the abrupt withdrawal of reproductive hormones after delivery (Schiller et al. 2015). A shared profile across different starting points could reflect equifinality in which distinct mechanisms, such as hormonal in PPD and varied pathways in non‐peripartum MDD, converge to a similar attachment pattern. The present cross‐sectional design though cannot distinguish these, which will require studies that model precipitating pathways directly. The divergences may in turn reflect the acute, event‐linked onset of PPD in samples recruited during a period of intense caregiving demand, in which women with otherwise secure attachment representations may nonetheless become depressed. Both interpretations remain tentative, as the PPD subgroups were small and the comparisons are between rather than within studies.

Clinical implications of the present findings suggest that attachment orientation may help to inform case formulation and treatment planning (Zilcha‐Mano 2021). Secure attachment is associated with better outcomes to psychotherapy (Levy et al. 2018). Increased attachment anxiety has been associated with a weaker therapeutic alliance, suggesting that attachment insecurity may influence early engagement with psychotherapy (Notsu et al. 2024). For individuals with anxious attachment, awareness of their rejection sensitivity, reassurance‐seeking and rumination may shape interventions to provide an early emphasis on emotion regulation, self‐criticism and maladaptive interpersonal expectations. In avoidant attachment, patients may mask distress, hinder help‐seeking and undermine therapeutic engagement. Approaches that gradually validate suppressed affect, bring about emotional awareness and tolerance of interpersonal closeness may help with engagement in treatment. Those with fearful attachment, marked by profound mistrust, approach‐avoidance interpersonal patterns and negative internal models of both self and others, may require an initial emphasis on safety, therapeutic alliance formation, shame reduction and relational stabilisation. However, whether attachment impacts on treatment outcomes is less consistent. Higher pre‐treatment avoidant attachment predicted greater reductions in depressive symptoms following Acceptance and Commitment Therapy (ACT) and CBT (A‐Tjak et al. 2020), though attachment anxiety and avoidance did not moderate treatment response to the Cognitive Behavioural Analysis System of Psychotherapy (CBASP) in patients with persistent depressive disorder (Diehm et al. 2025), suggesting that attachment may be more consistently associated with therapeutic processes, such as alliance formation and engagement, than with differential response to specific psychotherapies. Similar considerations may extend beyond MDD as a systematic review of bipolar disorder found insecure attachment to be associated with greater symptom burden, including depressive and anxiety symptoms, suicidality, hospitalisations, affective episodes and substance use (Suárez et al. 2026), suggesting that consideration of attachment is important across mental health disorders.

MDD is a clinically heterogeneous syndrome that may be comprised of distinct neuroanatomical dimensions associated with different clinical features and responses to treatment (Fu et al. 2019; Fu et al. 2024). The present findings suggest that attachment variation may contribute to this heterogeneity. Attachment‐related neural systems, including the hippocampus, amygdala, medial prefrontal cortex and anterior cingulate cortex, overlap with regions implicated in self‐referential processing, interpersonal threat, emotion regulation and stress responsivity in MDD (Bracht et al. 2022; Fu et al. 2024). In our previous work, one neuroanatomical profile was associated with greater childhood adversity and recent adverse life events, self‐harm and suicide attempts, poorer cognitive functioning and an immuno‐metabolic profile reflecting low‐grade inflammation with a comparable response to antidepressant or placebo treatment (Fu et al. 2024; Xiao et al. 2025). These features overlap with correlates of insecure attachment, including childhood maltreatment and adversity (Cyr et al. 2010; Lo et al. 2019; Widom et al. 2018), self‐harm and suicidality (Bifulco et al. 2002; Degnan et al. 2022), poorer emotion regulation and interpersonal functioning (Malik et al. 2015; Pietromonaco and Beck 2019) and stress‐related inflammatory and physiological dysregulation (Baumeister et al. 2016; Gouin et al. 2009; Jaremka et al. 2013). Insecure attachment has been linked to dysregulation of stress‐responsive biological systems, including hypothalamic–pituitary–adrenal, cardiovascular and immune pathways (Pietromonaco and Beck 2019). Anxious attachment is associated with altered cortisol production, reduced cellular immunity and elevated pro‐inflammatory cytokines (Jaremka et al. 2013), whereas avoidant attachment predicted increased interleukin‐6 responses to marital conflict (Gouin et al. 2009). Childhood maltreatment is associated with elevated adult inflammatory markers, including C‐reactive protein, interleukin‐6 and tumour necrosis factor‐α (Baumeister et al. 2016). Adult attachment insecurity partially mediates the association between childhood abuse or neglect and later depressive and anxious symptoms, with anxious attachment emerging as a mediator (Widom et al. 2018) and higher rates of enmeshed, fearful and angry‐dismissive attachment observed in adults with MDD with a history of childhood abuse (Bifulco et al. 2002). Together, these findings suggest that insecure attachment, early adversity and inflammation‐linked biological profiles may reflect a clinically meaningful subgroup in MDD.

Several limitations should be considered. Samples were predominantly female and drawn largely from Western settings, which limits generalisability, although the included studies came from a wide range of countries. Race and ethnicity were seldom reported, so conclusions about ethnic and cultural variation cannot be drawn. Most studies were cross‐sectional, limiting inference about whether attachment insecurity is a premorbid vulnerability, a consequence of depressive illness, or both. Healthy control groups were typically screened to exclude any psychiatric history, so the case–control contrasts may be larger than comparisons with an unselected community sample. Heterogeneity was substantial across analyses, reflecting differences in attachment measures and study designs. Subgroup analyses by instrument show reduced secure and elevated fearful attachment replicated on every measure contributing at least three studies, whereas the avoidant finding did not. Categorical classification is also cut‐off dependent as the same dimensional instrument can generate different proportions according to the threshold applied, which contributes to the heterogeneity of the prevalence estimates and limits their comparability with general population findings. The depression‐only studies reporting dimensional scores are summarised descriptively rather than converted into effect sizes against an assumed comparator as pooled control avoidance varied widely across instruments such that no control value was sufficiently stable as a reference. Publication bias was detected in the secure‐categorical and fearful‐categorical analyses, although trim‐and‐fill supported the robustness of both pooled effects.

In summary, this systematic review and meta‐analysis show that adults with MDD/PPD have markedly lower secure attachment than healthy controls and higher anxious, fearful and, to a lesser extent, avoidant attachment, which was instrument dependent. Approximately three in four adults with MDD/PPD have insecure attachment, with fearful, avoidant and anxious patterns of broadly comparable prevalence. These findings emphasise attachment as an important interpersonal dimension, with relevance to case formulation, therapeutic engagement and personalised treatment planning.

## Funding

L.X., V.C., A.M., R.W., G.S., V.L., M.O., M.H., A.L. and R.M. have nothing to declare. C.F. acknowledges support from the International Psychoanalytic Association (158102845), Milken Institute Baszucki Brain Research Fund (BD0000000029), National Institute of Mental Health (R01MH134236), National Institute for Health and Care Research (NIHR) Health Technology Assessment (NIHR165425), NIHR Efficacy and Mechanisms Evaluation (NIHR167361), Rosetrees Trust (CF2024M2116, CF20212104).

## Ethics Statement

This research did not involve any human participants or animal experimentation. Ethical approval was not required.

## Conflicts of Interest

The authors declare no conflicts of interest.

## Supporting information




**Table S1:** Attachment instruments used in the included studies.
**Table S2:** Equivalence mapping of attachment subtypes across categorical and continuous measures.
**Table S3:** Hartung‐Knapp sensitivity analysis (all outcomes).
**Table S4a:** Sensitivity analysis, contribution of depression‐only samples.
**Table S4b:** Pooled secure attachment against general‐population reference values.
**Table S5:** Dimensional attachment scores in depression‐only and case–control samples.
**Table S6:** Score ranges used for the POMP rescaling.
**Table S7:** Study summary.
**Table S8:** Participant characteristics for each study: race/ethnicity, depressive state.
**Table S9:** Quality assessment.
**Table S10a:** Continuous meta‐analysis scale‐specific exploratory subgroup analyses.
**Table S10b:** Categorical meta‐analysis scale‐specific exploratory subgroup analyses.
**Table S11a:** Categorical meta‐analysis MDD‐only subgroup.
**Table S11b:** Categorical meta‐analysis PPD‐only subgroup.
**Table S12a:** Continuous meta‐analysis MDD‐only subgroup.
**Table S12b:** Continuous meta‐analysis PPD‐only subgroup.
**Table S13a:** Univariate meta‐regression of moderators on categorical attachment outcomes (log OR).
**Table S13b:** Univariate meta‐regression of moderators on continuous attachment outcomes (Hedges' g).
**Table S14:** Pooled prevalence of attachment styles in MDD/PPD.
**Table S15:** Prevalence of attachment styles, MDD versus PPD subgroups.
**Figure S1:** Funnel plots of studies with continuous and categorical outcomes in four attachment types.
**Figure S2:** MDD‐only categorical forest plots.
**Figure S3:** PPD‐only categorical forest plots.
**Figure S4:** Pooled prevalence of attachment styles in clinically diagnosed MDD/PPD.

## Data Availability

Data sharing not applicable to this article as no datasets were generated or analysed during the current study.
