# Supplementary material for: Attachment Styles in Major Depressive Disorder: A Systematic Review and Meta‐Analysis
Source: Clin Psychol Psychother. 2026 Sep 30;33(5):e70343. doi: 10.1002/cpp.70343 (PMC13626741; doi:10.1002/cpp.70343)
Supplement: Supplementary file 1 [file CPP-33-e70343-s001.docx]

Attachment Styles in Major Depressive Disorder: A Systematic Review and Meta-Analysis PROSPERO CRD42017068580

**Supplementary Materials**

**Table of Contents**

Supplementary: Full Electronic Search Strategy	3

Supplementary: Reasons for exclusion of studies	5

Supplementary: Merging dimensions for multidimensional studies in meta-analysis for continuous measures	7

Supplementary: Qualitative synthesis of studies not included in the meta-analysis	8

Table S1. Attachment Instruments Used in the Included Studies	10

Table S2. Equivalence Mapping of Attachment Subtypes Across Categorical and Continuous Measures	13

Table S3. Hartung-Knapp Sensitivity Analysis (all outcomes)	14

Table S4a. Sensitivity Analysis, Contribution of Depression-Only Samples	15

Table S4b. Pooled Secure Attachment Against General-Population Reference Values	15

Table S5. Dimensional Attachment Scores in Depression-Only and Case-Control Samples	17

Table S6. Score Ranges Used for the POMP Rescaling	18

Table S7. Study Summary	22

Table S8. Participant characteristics for each study: race/ethnicity, depressive state	27

Table S9. Quality Assessment	28

Supplementary: Attachment findings by instrument	30

Table S10a. Continuous Meta-Analysis Scale-Specific Exploratory Subgroup Analyses	31

Table S10b. Categorical Meta-Analysis Scale-Specific Exploratory Subgroup Analyses	32

Table S11a. Categorical Meta-Analysis MDD-Only Subgroup	33

Table S11b. Categorical Meta-Analysis PPD-Only Subgroup	33

Table S12a. Continuous Meta-Analysis MDD-Only Subgroup	34

Table S12b. Continuous Meta-Analysis PPD-Only Subgroup	34

Table S13a. Univariate Meta-Regression of Moderators on Categorical Attachment Outcomes (log OR)	35

Table S13b. Univariate Meta-Regression of Moderators on Continuous Attachment Outcomes (Hedges’ g)	36

Table S14. Pooled Prevalence of Attachment Styles in MDD/PPD	37

Table S15. Prevalence of Attachment Styles, MDD versus PPD Subgroups	38

Figure S1. Funnel plots of studies with continuous and categorical outcomes in four attachment types	39

Figure S2. MDD-Only Categorical Forest Plots	43

Figure S3. PPD-Only Categorical Forest Plots	45

Figure S4. Pooled prevalence of attachment styles in clinically diagnosed MDD/PPD	47

#### Supplementary: Full Electronic Search Strategy

PROSPERO CRD42017068580; conducted and reported in accordance with PRISMA 2020. Query executed in each database, the filters applied, and the number of records retrieved is presented.

**1. Databases and approach**

Three electronic databases were searched from inception to the search date: 20 June 2026: PubMed, Scopus, and APA PsycINFO (via the Ovid platform). An identical set of keyword terms was applied across all three databases, combining a depression concept block with an attachment-style concept block using the Boolean operator AND. Terms were searched in the title, abstract, and keyword fields ([tiab] in PubMed; TITLE-ABS-KEY in Scopus; .ti,ab. in Ovid). In PubMed, the relevant Medical Subject Headings (MeSH) were additionally included. Reference lists of eligible articles were hand-searched for additional studies.

**2. Search strings, filters, and yields**

**PubMed (NCBI; inception-20 June 2026)**

("Depressive Disorder, Major"[Mesh] OR "Depression"[Mesh] OR "Depressive Disorder"[Mesh] OR depression[tiab] OR depressive[tiab] OR MDD[tiab] OR "major depress*"[tiab]) AND ("Object Attachment"[Mesh] OR "attachment style*"[tiab] OR "attachment pattern*"[tiab] OR "adult attachment"[tiab] OR "secure attachment"[tiab] OR "insecure attachment"[tiab] OR "fearful attachment"[tiab] OR "preoccupied attachment"[tiab] OR "dismissing attachment"[tiab] OR "anxious attachment"[tiab] OR "avoidant attachment"[tiab])

Filters/limits: English; Humans; Adult 18+; commentaries/editorials/single case reports excluded. Records retrieved: 2,877.

**Scopus (Elsevier, Advanced search; inception-20 June 2026)**

TITLE-ABS-KEY( ("major depress*" OR depression OR depressive OR MDD) AND ("attachment style*" OR "attachment pattern*" OR "adult attachment" OR "insecure attachment" OR "secure attachment" OR "anxious attachment" OR "avoidant attachment" OR "fearful attachment" OR "preoccupied attachment" OR "dismissing attachment") )

Filters/limits: Document type Article, Review; Language English; Subject area Psychology, Medicine, Nursing. Records retrieved: 2,118.

**APA PsycINFO (Ovid; inception-20 June 2026)**

(depression or depressive or MDD or "major depress*").ti,ab. AND ("attachment style*" or "attachment pattern*" or "adult attachment" or "insecure attachment" or "secure attachment" or "anxious attachment" or "avoidant attachment" or "fearful attachment" or "preoccupied attachment" or "dismissing attachment").ti,ab.

Filters/limits: English; peer-reviewed journal; adulthood (18+); human.

Records retrieved: 1,976.

**3. Records identified and de-duplication**

| **Stage** | **Records** |
| --- | --- |
| PubMed | 2,877 |
| Scopus | 2,118 |
| APA PsycINFO (Ovid) | 1,976 |
| **Total records identified** | **6,971** |
| Duplicate records removed before screening | 2,516 |
| Unique records screened (title/abstract) | 4,455 |

Records were combined and de-duplicated by matching on DOI and, where absent, on normalised title (2,516 duplicates removed). Of the 6,971 records, 819 were present in all three databases; the remainder were shared between database pairs or unique to a single database (PubMed only 1,682; PsycINFO only 653; Scopus only 446). A merged screening spreadsheet records, for each unique record, which database(s) retrieved it, providing a complete audit trail.

#### Supplementary: Reasons for exclusion of studies

Abapolnikova et al. (2026) analysed the same sample as Bernecker et al. (2016), which was retained, and was therefore excluded to avoid double-counting.

Bifulco et al. (2006) was a longitudinal re-report of the same 1990s community cohort as Bifulco et al. (2002), which was retained, and was therefore excluded to avoid double-counting.

**Bifulco et al. (2019)** reported the proportion of participants who exceeded a cut-off score for each attachment style, however, the criteria used did not enforce mutual exclusivity, meaning a single participant could meet the criteria for multiple attachment style. Since attachment styles are typically treated as mutually exclusive categories in categorical analyses, this is required for valid comparisons between groups. Including such data would risk inflating attachment style frequencies and biasing the results; thus, the study was excluded.

Conradi et al. (2018) reported on the same cohort as Conradi et al. (2009), which was retained, and was therefore not pooled to avoid double-counting.

Falkenström et al. (2024) was excluded because 47.1% of the sample had a comorbid personality disorder.

Fuhr et al. (2017) factor-analysed the Relationship Scales Questionnaire (RSQ) and reported effect sizes for their own data-driven factors rather than the conventional attachment styles, preventing meaningful comparison with the other included studies.

Galbally et al. (2023) reported attachment in a perinatal cohort in which the major depressive disorder subgroup could not be separated from the tabulated data, precluding extraction of MDD-only attachment scores.

Reis et al. (2004b) drew on the same University of Wollongong outpatient depression cohort as Reis et al. (2004a), which was retained; it was therefore not pooled, to avoid double-counting the same participants.

McMahon et al. (2005) administered the ASQ but reported only the subscales of Preoccupation with Relationships and Discomfort with Closeness, omitting the subscales Need for Approval and Relationships as Secondary. Since anxious attachment is defined as the sum of Preoccupation with Relationships and Need for Approval, and avoidant attachment as the sum of Discomfort with Closeness and Relationships as Secondary (Monteleone et al., 2018), the absence of these dimensions meant that comparable dimension scores could not be calculated.

Picardi et al. (2011) reported a mixed diagnostic sample in which the major depressive disorder subgroup (n = 9) was not separable from the tabulated Experiences in Close Relationships (ECR) data (n = 17), so MDD-only attachment data could not be extracted.

Veler-Poleg et al. (2025) was excluded because the majority of the sample (72.9%) had a comorbid personality disorder.

#### Supplementary: Merging dimensions for multidimensional studies in meta-analysis for continuous measures

Some studies employed multidimensional measures as continuous outcomes, such as the Attachment Style Questionnaire (ASQ; Feeney et al., 1994), the Attachment Style Interview (ASI; Bifulco et al., 2002), and the Adult Attachment Scale (Collins & Read, 1990), which measures three attachment dimensions. For studies implementing multidimensional measures, we calculated composite scores from these scales to reflect the two higher-order dimensions of attachment anxiety and attachment avoidance. Following the methodology from Monteleone et al. (2018), for ASQ, anxiety is calculated as a composite of need for approval and preoccupation with relationships, and avoidant is calculated as a composite score of discomfort with closeness and relationships as secondary. For AAS (Collins & Read, 1990), avoidant is calculated as a reversed composite score of the dimensions ‘close’ and ‘depend’. Means and SDs were pooled as standardised mean differences (Hedges’ g) between MDD and healthy control (HC) groups.

Six studies reported mean scores for the four attachment styles (i.e., secure, anxious-preoccupied, dismissing-avoidant, fearful) rather than following the conventional approach of classifying participants into discrete style categories. In these cases, we adopted Herstell et al.’s (2021) methodology, which combined anxious-preoccupied and attachment anxiety under the common label ‘anxious’, and dismissing-avoidant and attachment avoidance under the label ‘avoidant’. Herstell et al. (2021) demonstrated that combining attachment styles with attachment dimensions (anxiety, avoidance) yielded results comparable to analyses based solely on dimensions. Therefore, we treated the anxious-preoccupied style and attachment anxiety as equivalent indicators of an underlying “anxious” construct, and the dismissing-avoidant style and attachment avoidance as equivalent indicators of an “avoidant” construct. Fearful and secure constructs were retained under their original labels in both categorical and continuous analyses. Thus, in both categorical and continuous tests, we reported pooled estimates under the consistent labels secure, anxious, avoidant, and fearful.

#### Supplementary: Qualitative synthesis of studies not included in the meta-analysis

Five studies (Smith et al., 2012; Watson et al., 2014; Melnikov et al., 2021; Cho et al., 2025; Nowak et al., 2026) met the inclusion criteria, but did not provide extractable data for meta-analysis because attachment was reported as a predictor, correlate or model output rather than as an extractable between-group outcome. Of the 23 studies that contributed to the meta-analyses without a healthy-control group, eight reported mutually exclusive categorical attachment classifications and were eligible for the single-arm prevalence analyses (Tables S14–S12). The remaining 15 reported dimensional attachment anxiety and avoidance scores only. These cannot contribute a standardised mean difference, because the primary continuous analyses standardise each instrument against its healthy-control group. They are summarised individually in Table S7 above, and are additionally pooled descriptively on a Percentage of Maximum Possible metric in Tables S5 and S6, which shows that these samples do not differ from the patient arms of the case-control studies on either attachment anxiety or avoidance.

Smith et al. (2012) included 106 women with current major depression and a history of childhood sexual abuse to assess whether attachment anxiety and avoidance moderated associations between social maladjustment and death ideation. Neither attachment anxiety nor avoidance showed a significant main effect on death ideation.

Watson et al. (2014) included 55 patients with a current major depressive episode who received 16 sessions of cognitive-behavioural therapy or emotion-focused therapy. Across treatment, secure attachment increased and insecure attachment decreased with no significant treatment by time differences between the two groups. Greater client-perceived therapist empathy predicted improvements in both secure and insecure attachment, and these attachment changes statistically mediated associations between therapist empathy and a range of treatment outcomes, including depressive symptoms, dysfunctional attitudes, interpersonal problems, psychological distress and self-esteem.

Melnikov et al. (2021) examined attachment and facial-emotion recognition in 19 patients with depression and 20 healthy controls. Within the depressed group, greater attachment anxiety was associated with poorer recognition of angry facial expressions and better recognition of sad facial expressions.

Cho et al. (2025) used machine-learning regression models in 582 adults with depressive disorders to predict ECR-R anxious and avoidant attachment scores from psychiatric symptoms, early-life stress and heart-rate-variability measures. Predictive performance was greater for attachment anxiety than avoidance. Trait anxiety was the strongest predictor of anxious and avoidant ECR-R scores.

Nowak et al. (2026) included 74 outpatients with MDD during the initial phase of psychodynamic psychotherapy, assessing autobiographical memories elicited by attachment-related cues at baseline and after treatment. Attachment-cued autobiographical memory specificity increased during early treatment, while extended memories decreased. Higher baseline attachment anxiety was associated with less improvement in memory specificity, and higher attachment avoidance was associated with greater improvement.

#### Table S1. Attachment Instruments Used in the Included Studies

| **Abbrev.** | **Instrument** | **Format** | **Attachment constructs assessed and classification** | **k** | **Contributing studies** |
| --- | --- | --- | --- | --- | --- |
| ECR | Experiences in Close Relationships (Brennan, Clark, & Shaver, 1998) | 36-item self-report | Continuous attachment anxiety and avoidance; four-category classification derived by cross-classifying the two dimensions at a cut-off | 18 | A-Tjak 2020; Aceti 2012; Cho 2025; Conradi 2009; Craba 2023; Dagnino 2017; DiFilippo 2002; Gilbert 2009; Huh 2020; Marazziti 2007; Martinez 2023; Melnikov 2021; Nowak 2026; Özer 2015; Paradiso 2011; Picardi 2019; Smith 2012; Yotsidi 2024 |
| ECR-R | Experiences in Close Relationships–Revised (Fraley, Waller, & Brennan, 2000) | 36-item self-report | As ECR, with item-response-theory refined items | 4 | Bernecker 2016 (with RSQ); Ihme 2022; Martucci 2021; Oon-Arom 2019 |
| ECR-RS | Experiences in Close Relationships–Relationship Structures (Fraley et al., 2011) | 9-item self-report | Attachment anxiety and avoidance, rated separately for each relationship target | 1 | Cheng 2025 |
| ECR-II | Experiences in Close Relationships Inventory–II; Turkish adaptation of the ECR-R (Selçuk, Günaydın, Sümer, & Uysal, 2005) | Self-report | Attachment anxiety and avoidance | 1 | Özaslan 2025 |
| AAS | Adult Attachment Scale (Collins & Read, 1990) | 18-item self-report | Close, Depend and Anxiety subscales; classification into secure, anxious and avoidant styles | 7 | Bartolomei 2020; Golshani 2021; Grunebaum 2010; Kani 2023; Karabekiroglu 2011; Koelkebeck 2017; Levitan 2009 |
| AAS-R | Adult Attachment Scale–Revised (Schmidt, Muehlan, & Brähler, 2016) | 18-item self-report | As AAS | 1 | Bracht 2022 |
| RAAS | Revised Adult Attachment Scale (Collins, 1996) | 18-item self-report | Close, Depend and Anxiety subscales | 2 | Reinecke 2001; Zhang 2023 |
| AASS | Adult Attachment Style Scale (Mikulincer, Florian, & Tolmacz, 1990) | Self-report | Secure, anxious and avoidant styles | 1 | Demıray 2025 |
| RQ | Relationship Questionnaire (Bartholomew & Horowitz, 1991) | Four-paragraph self-report | Forced-choice classification into secure, preoccupied, dismissing or fearful, plus continuous ratings of each of the four prototypes | 6 | Huang 2019; Niolu 2016; Reis 2004; Troxel 2007; Whiffen 2001; Wilkinson 2010 |
| RSQ | Relationship Scales Questionnaire (Griffin & Bartholomew, 1994) | 30-item self-report | Continuous scores on the same four prototypes as the RQ; categorical classification by the highest prototype score | 6 | Bernecker 2016 (with ECR-R); Çelikbaş 2022; Fuhr 2017; König 2026; McBride 2006; Strecker 2025 |
| ASQ | Attachment Style Questionnaire (Feeney, Noller, & Hanrahan, 1994) | 40-item self-report | Five subscales: Confidence, Discomfort with Closeness, Need for Approval, Preoccupation with Relationships, Relationships as Secondary | 4 | Cacciola 2020; Costa 2009; McMahon 2005; Watson 2014 |
| ASI | Attachment Style Interview (Bifulco et al., 2002) | Semi-structured, investigator-based interview | Mutually exclusive categories: secure, enmeshed, fearful, angry-dismissive and withdrawn (the latter two combined as avoidant in the present analyses) | 5 | Bifulco 2002; Ikeda 2014; Kwon 2017; Nonnenmacher 2016; Spence 2020 |
| AAI | Adult Attachment Interview (George, Kaplan, & Main, 1985) | Semi-structured interview, transcript coded by trained raters | Mutually exclusive classifications: secure-autonomous, dismissing, preoccupied and unresolved/cannot classify (Main, Goldwyn, & Hesse, 2002) | 3 | Murray 2006; Reiner 2016; Santona 2015 |
| AAP | Adult Attachment Projective Picture System (George & West, 2001) | Projective picture-response task, transcript coded by trained raters | The same four classifications as the AAI | 1 | Bauriedl-Schmidt 2017 |
| AAPR | Adult Attachment Prototype Rating (Strauss, Lobo-Drost, & Pilkonis, 1999) | Interview-based prototype rating | Secure, dismissing, preoccupied and fearful prototypes | 1 | Strauss 2006 |
| VASQ | Vulnerable Attachment Style Questionnaire (Bifulco et al., 2003) | 22-item self-report screening measure | Insecurity and Proximity-seeking scales; screens for attachment insecurity rather than assigning mutually exclusive styles | 1 | Bifulco 2019 |

Attachment instruments used across all 61 studies included in the systematic review. Fifteen distinct instruments were used, giving 62 instrument uses: Bernecker (2016) administered both the ECR-R and the RSQ, and the ECR-II used by Özaslan (2025) is the Turkish adaptation of the ECR-R (Selçuk et al., 2005), shown on its own row for transparency but counted as the ECR-R. Self-report questionnaires account for 52 of these uses and interview-based or rater-coded measures for the remainder. Instruments differ in whether they yield continuous dimensions (attachment anxiety and avoidance), continuous prototype ratings, or mutually exclusive categorical classifications; where a categorical outcome was derived from a dimensional instrument, the classification depends on the cut-off applied by the original authors, which is one source of the heterogeneity observed in the categorical and prevalence analyses. Only studies reporting mutually exclusive categorical classifications contributed to the categorical case-control and single-arm prevalence analyses; the VASQ screens for insecurity rather than assigning mutually exclusive styles and therefore contributed only to the secure-versus-insecure comparison. Study-level instrument assignments are also given in Table 1 of the main text.

#### Table S2. Equivalence Mapping of Attachment Subtypes Across Categorical and Continuous Measures

| **Common Framework** | **Categorical Subtypes** | **Continuous Dimensions** | **Description/Key Characteristics** |
| --- | --- | --- | --- |
| Secure | Secure  /Autonomous | Security | Coherent narratives; trust in self/others; effective emotion regulation; balanced proximity-seeking. |
| Preoccupied/ Anxious | Preoccupied /Enmeshed /Anxious-Resistant | Anxiety | Hyperactivating strategies; fear of abandonment; rumination; heightened emotional entanglement; negative self-view (unlovable) with variable views of others. |
| Dismissive/ Avoidant | Dismissive  /Avoidant | Avoidance | Deactivating strategies; emotional suppression; idealization/derogation of relationships; positive self-view with negative views of others (unreliable). |
| Fearful/ Disorganised | Fearful  /Disorganised  /Unresolved | Fearful/ Disorganisation (high anxiety + high avoidance) | Collapse of strategies; trauma-linked; emotional volatility; negative views of self (unlovable) and others (unreliable/unloving); dissociative responses. |

**Table S3. Hartung-Knapp Sensitivity Analysis (all outcomes)**

As a sensitivity analysis, all pooled estimates were re-computed using the Hartung-Knapp-Sidik-Jonkman adjustment (REML between-study variance; confidence intervals and p values derived from the t-distribution with k-1 degrees of freedom), which provides more conservative inference under heterogeneity. Results were largely unchanged. Reduced secure attachment and elevated anxious attachment in MDD remained significant for both the continuous and categorical outcomes. The avoidant elevation was borderline under the more conservative Hartung-Knapp intervals, remaining marginally significant on the continuous dimension (Hedges’ g = 0.41, 95% CI [0.01, 0.81], p = .047) but not on the categorical comparison (OR = 1.42, 95% CI [0.99, 2.04], p = .053); the continuous avoidant effect was also measure-dependent (ECR-only g = 0.93). Fearful attachment remained non-significant on the continuous dimension (g = 0.35, 95% CI [−0.18, 0.87], p = .14) but significant on the categorical comparison (OR = 3.00, p < .001).

| **Outcome** | **k** | **Estimate** | **HK 95% CI** | **p (HK)** | **I²** |
| --- | --- | --- | --- | --- | --- |
| Secure, continuous (g) | 7 | −0.46 | [−0.85, −0.07] | .029 | 86% |
| Anxious, continuous (g) | 15 | 0.63 | [0.38, 0.89] | <.001 | 81% |
| Avoidant, continuous (g) | 15 | 0.41 | [0.01, 0.81] | .047 | 92% |
| Fearful, continuous (g) | 5 | 0.35 | [−0.18, 0.87] | .14 | 86% |
| Secure, categorical (OR) | 21 | 0.20 | [0.15, 0.27] | <.001 | 43% |
| Anxious/preoccupied, cat. (OR) | 16 | 2.46 | [1.60, 3.78] | <.001 | 43% |
| Avoidant/dismissive, cat. (OR) | 16 | 1.42 | [0.99, 2.04] | .053 | 31% |
| Fearful, categorical (OR) | 12 | 3.00 | [1.84, 4.87] | <.001 | 38% |

Note. Estimate = Hedges’ g for continuous (standardised mean difference) outcomes and odds ratio for categorical outcomes, MDD versus healthy controls. HK = Hartung-Knapp-Sidik-Jonkman; between-study variance estimated by REML; confidence intervals and p values from the t-distribution (k−1 df).

#### Table S4a. Sensitivity Analysis, Contribution of Depression-Only Samples

| **Attachment style** | **Case-control patient arms only, k — % [95% CI]** | **Including depression-only samples, k — % [95% CI]** | **Difference** |
| --- | --- | --- | --- |
| Secure | 21 — 25.4 [20.8, 30.7] | 29 — 24.1 [19.8, 28.9] | -1.3 pp |
| Anxious / preoccupied | 18 — 21.8 [17.0, 27.5] | 25 — 23.0 [17.8, 29.2] | +1.2 pp |
| Avoidant / dismissive | 18 — 21.7 [15.2, 30.0] | 25 — 23.7 [18.0, 30.5] | +2.0 pp |
| Fearful | 15 — 29.4 [24.2, 35.2] | 20 — 26.9 [21.9, 32.5] | -2.5 pp |

Sensitivity analysis comparing the pooled prevalence estimated from the patient arms of the case-control studies alone with the estimate obtained after adding depression-only samples. Each estimate shifts by no more than 2.5 percentage points, indicating that the pooled prevalences are not driven by the inclusion of single-arm studies. pp = percentage points.

#### Table S4b. Pooled Secure Attachment Against General-Population Reference Values

| **Reference prevalence** | **Odds ratio** | **95% CI** | **z** | **I² (%)** |
| --- | --- | --- | --- | --- |
| 40% | 0.49 | [0.38, 0.63] | -5.63 | 64.7 |
| 45% | 0.40 | [0.31, 0.51] | -7.24 | 65.1 |
| 50% | 0.33 | [0.25, 0.42] | -8.82 | 65.2 |
| 55% (Shaver & Clark, 1994) | 0.27 | [0.21, 0.34] | -10.39 | 65.1 |
| 59% (Mickelson et al., 1997) | 0.23 | [0.18, 0.29] | -11.67 | 64.8 |
| 65% | 0.17 | [0.14, 0.22] | -13.65 | 64.0 |

Pooled odds of secure attachment in clinically diagnosed MDD/PPD relative to a range of general-population reference values, across the same 29 samples (1,905 patients) as Table S14, in which the pooled prevalence of secure attachment was 24.9%. Each study’s observed count was compared with a reference arm of the same size at the stated prevalence, and the resulting odds ratios pooled by random-effects meta-analysis. The reduction remains statistically significant against any reference value above 29.4%. The comparison is presented as a range because no single population value is strictly commensurate with a pooled estimate drawn from several instruments: categorical classification of attachment depends on the measure used and the cut-off applied, and the commonly cited figures of 55% (Shaver & Clark, 1994) and 59% (Mickelson et al., 1997) derive from three-category forced-choice self-report measures rather than from the four-category schemes used by most contributing studies. For comparison, the pooled healthy-control prevalence of secure attachment in the present meta-analysis was 63.4%. The reference was varied from 40% to 65% to span these values with a margin either side.

#### Table S5. Dimensional Attachment Scores in Depression-Only and Case-Control Samples

| **Dimension** | **Sample set** | **k** | **Pooled POMP, % of scale range [95% CI]** | **I² (%)** | **τ²** |
| --- | --- | --- | --- | --- | --- |
| Attachment anxiety | All MDD/PPD patient arms | 32 | 46.9 [43.2, 50.6] | 97.0 | 106.7 |
|  | Depression-only studies | 17 | 46.8 [42.2, 51.4] | 97.2 | 87.5 |
|  | Patient arms of case-control studies | 15 | 47.0 [40.9, 53.1] | 96.2 | 136.7 |
|  | Healthy-control arms (context only) | 15 | 34.2 [27.3, 41.1] | 98.3 | 180.9 |
|  | Depression-only vs case-control patient arms |  | difference -0.2 points | Qᵇ = 0.00 | p = 0.97 |
| Attachment avoidance | All MDD/PPD patient arms | 29 | 46.0 [41.5, 50.5] | 96.6 | 147.0 |
|  | Depression-only studies | 14 | 45.8 [40.9, 50.8] | 95.9 | 82.5 |
|  | Patient arms of case-control studies | 15 | 46.0 [38.4, 53.6] | 96.7 | 217.3 |
|  | Healthy-control arms (context only) | 15 | 39.0 [28.9, 49.2] | 99.4 | 398.7 |
|  | Depression-only vs case-control patient arms |  | difference -0.2 points | Qᵇ = 0.00 | p = 0.97 |

Dimensional attachment scores rescaled to Percentage of Maximum Possible (POMP), POMP = 100 × (M − minimum) / (maximum − minimum), using the score range of the instrument as scored in each study (Table S6), and pooled by inverse-variance random-effects meta-analysis with REML estimation of the between-study variance. No healthy-control value is assumed anywhere in this analysis: it summarises the distribution of attachment scores within each set of samples, and is the dimensional counterpart of the categorical prevalence analyses in Tables S14–S12a. k = number of contributing samples; Levitan (2009) contributes two non-overlapping subgroups (atypical and melancholic). Subgroup differences were tested by Q between with 1 degree of freedom. Depression-only and case-control samples did not differ on either dimension, and the difference did not exceed 2.5 POMP points in any sensitivity analysis (omitting Levitan's two subgroups, omitting McMahon (2004), omitting all studies whose score range required checking against the source paper, or adding Cheng (2025) under the item allocation stated in that paper). Because these are pooled raw scores rather than effect sizes, heterogeneity is necessarily high and the pooled values are descriptive; they are not comparable with the standardised mean differences reported in the main analyses. The healthy-control rows are given for context only and were not used as a reference: pooled control avoidance ranged from 28.9 to 49.2 POMP points across instruments (I² = 99.4%), so no single control value was sufficiently stable to serve as a comparator.

#### Table S6. Score Ranges Used for the POMP Rescaling

| **Study** | **Instrument** | **Range** | **Basis for the range** | **Sample** | **n** | **Anxiety** | **Avoidance** |
| --- | --- | --- | --- | --- | --- | --- | --- |
| A-Tjak 2020 | ECR | 18–126 | instrument specification | Depression-only | 82 | 47.8 | 37.9 |
| Bartolomei 2020 | AAS | 1–5 | instrument specification | Depression-only | 234 | 49.3 | 40.5 |
| Bernecker 2016 | ECR-R | 1–7 | instrument specification | Depression-only | 69 | 45.2 | 39.3 |
| Dagnino 2017 | ECR | 1–7 | instrument specification | Depression-only | 70 | 60.7 | 41.3 |
| DeFilippo 2002 | ECR | 1–7 | instrument specification | Depression-only | 84 | 58.7 | 40.8 |
| Gilbert 2009 | ECR | 18–126 | instrument specification | Depression-only | 62 | 58.0 | 46.6 |
| Grunebaum 2010 | AAS (Simpson, 1990) | 5–35 | source paper | Depression-only | 132 | 50.9 | — |
| Huang 2019 | RQ (Chinese) | 6–36 | source paper | Depression-only | 17 | 44.0 | 65.3 |
| Huh 2019 | ECR | 18–126 | instrument specification | Depression-only | 294 | 31.4 | 34.2 |
| Ihme 2022 | ECR-R | 18–126 | instrument specification | Depression-only | 96 | 54.2 | 36.3 |
| Levitan 2009 (atypical) | AAS | 6–30 | instrument specification | Depression-only | 32 | 57.5 | 63.3 |
| Levitan 2009 (melancholic) | AAS | 6–30 | instrument specification | Depression-only | 79 | 47.1 | 56.3 |
| Martinez 2023 | ECR | 1–7 | instrument specification | Depression-only | 178 | 36.7 | 53.3 |
| Oon-Arom 2019 | ECR-R | 1–7 | instrument specification | Depression-only | 119 | 30.0 | 48.8 |
| Reinecke 2001 | RAAS | 6–30 | instrument specification | Depression-only | 54 | 32.9 | — |
| Yotsidi 2024 | ECRS | 1–7 | instrument specification | Depression-only | 273 | 44.5 | 43.7 |
| Zhang 2023 | RAAS | 6–30 | instrument specification | Depression-only | 139 | 48.2 | — |
| Bracht 2022 | AAS-R | 6–30 | instrument specification | MDD/PPD (case-control) | 48 | 50.0 | — |
| Costa 2009 | ASQ | 8.5–51 | source paper | MDD/PPD (case-control) | 93 | 51.7 | 39.5 |
| Craba 2023 | ECR | 18–126 | source paper | MDD/PPD (case-control) | 51 | 50.3 | 33.0 |
| Fuhr 2017 | RSQ (German) | 1–5 | instrument specification | MDD/PPD (case-control) | 61 | 40.2 | 35.5 |
| Karabekiroglu 2011 | AAS | 6–30 | instrument specification | MDD/PPD (case-control) | 32 | 20.3 | 34.5 |
| Koelkebeck 2017 | AAS | 6–30 | instrument specification | MDD/PPD (case-control) | 38 | 39.2 | 56.7 |
| König 2026 | RSQ | 1–5 | instrument specification | MDD/PPD (case-control) | 76 | — | 47.2 |
| Martucci 2021 | ECR-R | 18–126 | instrument specification | MDD/PPD (case-control) | 46 | 44.5 | 30.0 |
| McBride 2006 | RSQ | 1–5 | instrument specification | MDD/PPD (case-control) | 75 | 54.0 | 58.2 |
| McMahon 2004 | ASQ | 10–60 | inferred; see note | MDD/PPD (case-control) | 62 | 45.4 | 49.5 |
| Ozer 2015 | ECR | 18–126 | instrument specification | MDD/PPD (case-control) | 62 | 63.0 | 58.4 |
| Picardi 2019 | ECR | 18–126 | instrument specification | MDD/PPD (case-control) | 21 | 46.2 | 43.2 |
| Reis 2004a | RQ | 1–100 | source paper | MDD/PPD (case-control) | 71 | 44.4 | 49.1 |
| Whiffen 2001 | RQ | 1–7 | instrument specification | MDD/PPD (case-control) | 52 | 41.7 | 33.3 |
| Wilkinson 2010 | RQ | 1–7 | instrument specification | MDD/PPD (case-control) | 47 | 39.5 | 34.0 |
| Çelikbaş 2022 | RSQ | 1–5 | instrument specification | MDD/PPD (case-control) | 124 | 73.2 | 87.5 |
| Bracht 2022 | AAS-R | 6–30 | instrument specification | Healthy control | 18 | 16.7 | — |
| Costa 2009 | ASQ | 8.5–51 | source paper | Healthy control | 192 | 40.1 | 33.2 |
| Craba 2023 | ECR | 18–126 | source paper | Healthy control | 60 | 33.3 | 18.1 |
| Fuhr 2017 | RSQ (German) | 1–5 | instrument specification | Healthy control | 60 | 35.5 | 23.8 |
| Karabekiroglu 2011 | AAS | 6–30 | instrument specification | Healthy control | 174 | 18.6 | 24.9 |
| Koelkebeck 2017 | AAS | 6–30 | instrument specification | Healthy control | 40 | 15.4 | 80.8 |
| König 2026 | RSQ | 1–5 | instrument specification | Healthy control | 76 | — | 31.8 |
| Martucci 2021 | ECR-R | 18–126 | instrument specification | Healthy control | 28 | 27.8 | 10.4 |
| McBride 2006 | RSQ | 1–5 | instrument specification | Healthy control | 151 | 50.7 | 67.5 |
| McMahon 2004 | ASQ | 10–60 | inferred; see note | Healthy control | 38 | 38.0 | 38.2 |
| Ozer 2015 | ECR | 18–126 | instrument specification | Healthy control | 60 | 41.9 | 52.0 |
| Picardi 2019 | ECR | 18–126 | instrument specification | Healthy control | 21 | 41.3 | 23.6 |
| Reis 2004a | RQ | 1–100 | source paper | Healthy control | 245 | 41.6 | 50.1 |
| Whiffen 2001 | RQ | 1–7 | instrument specification | Healthy control | 60 | 31.7 | 30.0 |
| Wilkinson 2010 | RQ | 1–7 | instrument specification | Healthy control | 68 | 16.5 | 36.2 |
| Çelikbaş 2022 | RSQ | 1–5 | instrument specification | Healthy control | 144 | 62.7 | 65.0 |

Score range applied to each study and the resulting POMP value; the Anxiety and Avoidance columns are POMP values, as percentages of the instrument’s score range. “Source paper” means the range, or the item count and response scale from which it follows, is given in the paper itself; “instrument specification” means the standard specification for that instrument was applied and has not yet been checked against the paper. Standard specifications used: ECR, ECR-R and ECRS, 18 items per subscale rated 1–7 (summed 18–126, item mean 1–7); Collins and Read Adult Attachment Scale and its revision, 6 items per subscale rated 1–5 (summed 6–30, item mean 1–5); Relationship Scales Questionnaire, item mean on a 5-point scale; Relationship Questionnaire prototype ratings on a 7-point scale. Two entries required judgement. McMahon (2004) describes its two Attachment Style Questionnaire subscales as comprising 13 items in total, which is not compatible with its own reported means; the standard item counts for Discomfort with Closeness (10 items) and Preoccupation with Relationships (8 items) were used instead, and give control values closely matching those of Costa (2009) on the same subscales. Cheng (2025) is omitted: under the item allocation stated in that paper the reported standard deviations exceed what is arithmetically possible for the implied score ranges, and the scoring could not be reconstructed. Grunebaum (2010) used Simpson’s (1990) 13-item Adult Attachment Scale rather than the Collins and Read measure, and only its anxious factor was available. Levitan (2009) contributes two non-overlapping subgroups. Em dash indicates that the dimension was not reported in an extractable form.

#### Table S7. Study Summary

Setting, diagnostic basis, depression measure, current-episode status, and key attachment findings for each included study.

| **Study** | **Setting** | **Type** | **Diagnosis (criteria; instrument)** | **Depression scale (inclusion threshold)** | **Episode status** | **Key attachment findings** |
| --- | --- | --- | --- | --- | --- | --- |
| A-Tjak (2020) | Community | MDD | DSM-IV; SCID | QIDS | Current episode | Dimensional (MDD only): anxious, avoidant scores reported |
| Aceti (2012) | Community | PPD | DSM-IV; SCID | EPDS (EPDS ≥ 12) | Current episode | Categorical (MDD vs HC): secure lower in MDD (45% vs 82%); insecure styles more common in MDD |
| Bartolomei (2020) | Community | MDD | DSM-IV; SCID | MADRS (MADRS > 7) | Current episode | Dimensional (MDD only): anxious, avoidant scores reported |
| Bauriedl-Schmidt (2017) | Hospital | MDD | DSM-IV; SCID | HRSD-17 | Current episode | Categorical (MDD vs HC): secure lower in MDD (5% vs 56%); insecure styles more common in MDD |
| Bernecker (2016) | Community | MDD | DSM-IV; SCID | HRSD-17 (HRSD > 16) | Current episode | Dimensional (MDD only): anxious, avoidant scores reported |
| Bilfuco (2002) | Community | MDD | DSM-III-R; PSE | PSE (PSE > 4 symptoms) | Current episode | Categorical (MDD only; depressed subsample n=89): secure 15% (13/89), preoccupied 18% (16), dismissive 34% (30), fearful 34% (30). Non-depressed women not used as controls (drawn from the same high-risk community sample). |
| Bilfuco (2019) | Community | MDD | DSM-5; Clinical interview | GHQ-12 (GHQ > 5) | Current episode | Categorical (MDD vs HC): secure lower in MDD (44% vs 77%); insecure styles more common in MDD |
| Bracht (2022) | Mixed | MDD | DSM-IV; MINI | HRSD-21 | Current episode | Dimensional (MDD vs HC): anxious higher in MDD |
| Cacciola (2020) | Community | PPD | DSM-IV; PRIME-MD | EPDS (EPDS ≥ 11.5) | Current episode | Categorical (MDD only): predominant style secure (41%) |
| Celikbas (2022) | Community | MDD | DSM-5; Clinical interview | PHQ-9 | Range of symptom severity | Dimensional (MDD vs HC): anxious higher, avoidant higher, fearful higher in MDD |
| Cheng (2025) | Hospital | MDD | ICD-10; Clinical interview | HDRS-17 | Current episode | Dimensional (MDD only): anxious, avoidant scores reported |
| Cho (2025) | Mixed | MDD | DSM-5; Clinical interview | HRSD-17 | Range of symptom severity | Trait anxiety was the strongest ECR-R predictor. |
| Conradi (2009) | Community | MDD | DSM-IV; CIDI | BDI | Current episode | Categorical (MDD only): predominant style secure (43%) |
| Costa (2009) | Community | MDD | DSM-IV; SCID | HRSD-17 | Range of symptom severity | Dimensional (MDD vs HC): secure higher, anxious higher, avoidant higher in MDD |
| Craba (2023) | Community | MDD | DSM-V-TR; Clinical interview | HRSD-17 | Mild symptom severity | Dimensional (MDD vs HC): anxious higher, avoidant higher in MDD \| Categorical (MDD vs HC): secure lower in MDD (27% vs 90%); insecure styles more common in MDD |
| Dagnino (2017) | Community | MDD | ICD-10; Clinical interview | BDI/OQ | Current episode | Dimensional (MDD only): anxious, avoidant scores reported |
| DiFilippo (2002) | Hospital | MDD | DSM-IV; SCID-I | BDI | Current episode | Higher depressive symptoms associated with more insecure attachment to parents and to current romantic partner (dimensional). |
| Demıray (2025) | Community | MDD | DSM-5; Clinical interview | HRSD-17 (HRSD > 8) | Current episode | Categorical (MDD vs HC): secure lower in MDD (20% vs 52%); insecure styles more common in MDD |
| Fuhr (2017) | Community | MDD | DSM-IV; SCID | HRSD-17 (HRSD > 7) | Remission | Dimensional (MDD vs HC): anxious higher, avoidant higher in MDD |
| Gilbert (2009) | Hospital | MDD | ICD; Clinical interview | DASS-21 depression | Current episode | Dimensional (MDD only): anxious, avoidant scores reported |
| Golshani (2021) | Community | MDD | DSM-V; Clinical interview | BDI-II (BDI > 30) | Current episode | Categorical (MDD only): predominant style preoccupied (72%) |
| Gruunebaum (2010) | Hospital | MDD | DSM-IV; SCID | HRSD-17 (HRSD > 16) | Current episode | Dimensional (MDD only): anxious scores reported |
| Huang (2019) | Community | MDD | DSM-IV-TR; MINI | HAMD | Current episode | Dimensional (MDD only): secure, anxious, avoidant, fearful scores reported \| Categorical (MDD only): predominant style secure (35%) |
| Huh (2020) | Community | MDD | DSM-IV; MINI | BDI | Current episode | Dimensional (MDD only): anxious, avoidant scores reported |
| Ihme (2022) | Community | MDD | DSM-IV; Clinical interview | HRSD-17 (HRSD score > 19) | Current episode | Dimensional (MDD only): anxious, avoidant scores reported |
| Ikeda (2014) | Community | PPD | DSM-IV; MINI | EPDS | Current episode | Categorical (MDD vs HC): secure lower in MDD (38% vs 65%); insecure styles more common in MDD |
| Kani (2023) | Community | MDD | DSM-5; SCID | BDI (NR) | Range of symptom severity | Categorical (MDD vs HC): secure lower in MDD (32% vs 72%); insecure styles more common in MDD |
| Karabekiroglu (2011) | Community | MDD | DSM-IV; SCID | BDI | Range of symptom severity | Dimensional (MDD vs HC): secure lower, anxious higher, avoidant higher in MDD |
| Koelkeback (2017) | Hospital | MDD | DSM-IV; SCID | HRSD-21 (HRSD > 8) | Range of symptom severity | Dimensional (MDD vs HC): anxious higher, avoidant lower in MDD |
| König (2026) | Community | MDD | DSM-5; SCID-5-CV | HRSD-17 / BDI-II (BDI-II ≥ 9) | Current episode | Dimensional (MDD vs HC): avoidant higher in MDD |
| Kwon (2017) | Community | MDD | DSM-IV; SCID | BDI-II | Range of symptom severity | Categorical (MDD vs HC): secure lower in MDD (46% vs 87%); insecure styles more common in MDD |
| Levitan (2009) | Community | MDD | DSM-IV; SCID | HRSD-17 | Range of symptom severity | Dimensional (MDD only): secure, anxious, avoidant scores reported \| Dimensional (MDD only): secure, anxious, avoidant scores reported |
| Marazziti (2007) | Community | MDD | DSM-IV; SCID |  | Range of symptom severity | Categorical (MDD vs HC): secure lower in MDD (18% vs 60%); insecure styles more common in MDD |
| Martinez (2023) | Community | MDD | ICD-10; Clinical interview | PHQ-9 | Current episode | Dimensional (MDD only): anxious, avoidant scores reported |
| Martucci (2021) | Community | PPD | DSM-V; Clinical interview | EPDS (EPDS > 8) | Current episode | Dimensional (MDD vs HC): anxious higher, avoidant higher in MDD \| Categorical (MDD vs HC): secure lower in MDD (37% vs 86%); insecure styles more common in MDD |
| McBride (2006) | Community | MDD | DSM-IV; SCID | BDI-II | Current episode | Dimensional (MDD vs HC): secure lower, anxious higher, avoidant lower, fearful lower in MDD |
| McMahon (2004) | Community | PPD | DSM-IV; CIDI | CES-D | Range of symptom severity | Dimensional (MDD vs HC): anxious higher, avoidant higher in MDD |
| Melnikov (2021) | Community | MDD | ICD-10; Clinical interview | BDI | Current episode | Narrative: within MDD, higher anxious attachment linked to altered facial-emotion recognition (poorer angry, better sad detection); associations absent in HC. |
| Murray (2006) | Community | PPD | DSM-IV; SADS, SCID | EPDS, MFQ (EPDS > 12) | Range of symptom severity | Categorical (MDD vs HC): secure lower in MDD (20% vs 55%); insecure styles more common in MDD |
| Niolu (2016) | Community | MDD | DSM-IV/ICD-10; MINI Plus | BDI | Range of symptom severity | Categorical (MDD vs HC): secure lower in MDD (20% vs 27%); insecure styles more common in MDD |
| Nonnenmacher (2016) | Community | PPD | DSM-IV; SCID | (NA) | Range of symptom severity | Categorical (MDD vs HC): secure lower in MDD (29% vs 68%); insecure styles more common in MDD |
| Nowak (2026) | Community | MDD | DSM-IV; SCID-I/II | BDI-II | Range of symptom severity | Narrative: higher baseline attachment anxiety predicted less improvement in autobiographical-memory specificity; higher avoidance and greater reduction in affective suppression linked to improvement. |
| Oon-Arom (2019) | Community | MDD | DSM-IV; MINI | (NA) | Current episode | Dimensional (MDD only): anxious, avoidant scores reported |
| Özaslan (2025) | Community | MDD | DSM-5; Clinical interview | BDI | Current episode | Categorical/dimensional (MDD vs HC): patients showed more insecure attachment and maladaptive schemas than controls — verify specific dimensions. |
| Özer (2015) | Community | MDD | DSM-IV-TR; SCID | HRSD-17 | Range of symptom severity | Dimensional (MDD vs HC): anxious higher, avoidant higher in MDD \| Categorical (MDD vs HC): secure lower in MDD (6% vs 40%); insecure styles more common in MDD |
| Paradiso (2011) | Community | MDD | DSM-IV-TR; SCID | HRSD-17 | Range of symptom severity | Categorical (MDD vs HC): secure lower in MDD (29% vs 60%); insecure styles more common in MDD |
| Picardi (2019) | Community | MDD | DSM-IV-TR; SCID | BPRS-24 | Current episode | Dimensional (MDD vs HC): anxious higher, avoidant higher in MDD |
| Reinecke (2001) | Community | MDD | DSM-IV; SCID | BDI | Current episode | Dimensional (MDD only): anxious scores reported |
| Reiner^ (2016) | Hospital | MDD | DSM-IV; SCID | PHQ-9 | Current episode | Categorical (MDD vs HC): secure lower in MDD (12% vs 64%); insecure styles more common in MDD |
| Reis (2004) | Community | MDD | DSM-IV; SCID | BDI | Current episode | Dimensional (MDD vs HC): secure lower, anxious higher, avoidant lower, fearful higher in MDD |
| Santona (2015) | Community | PPD | DSM-5; SCID | (NA) | Current episode | Categorical (MDD vs HC): secure lower in MDD (33% vs 60%); insecure styles more common in MDD |
| Smith (2012) | Community | MDD | DSM-IV; SCID | HRSD-17 | Current episode | Narrative: in depressed women with a history of childhood sexual abuse, insecure attachment and social maladjustment were associated with death ideation. |
| Spence (2020) | Community | MDD | DSM-IV; SCID | (NA) | Current episode | Categorical (MDD only): predominant style dismissive (67%) |
| Strauss (2006) | Community | MDD | ICD-10; Clinical interview | (NA) | Current episode | Categorical (MDD only): predominant style preoccupied (48%) |
| Strecker (2025) | Hospital | MDD | DSM-IV; Mini-DIPS | BDI-II | Current episode | Categorical (MDD vs HC): secure lower in MDD (21% vs 51%); insecure styles more common in MDD |
| Troxel (2007) | Community | MDD | DSM-IV; SADS or SCID | HRSD-17 (HRSD ≥ 15) | Current episode | Categorical (MDD only): predominant style fearful (39%) |
| Watson (2014) | Community | MDD | DSM-IV; Clinical interview | BDI | Current episode | Narrative: perceived therapist empathy was associated with positive change in clients’ attachment style during treatment (mediation model). |
| Whiffen (2001) | Community | MDD | DSM-III-R; SADS | BDI | Range of symptom severity | Dimensional (MDD vs HC): secure lower, anxious higher, avoidant higher, fearful higher in MDD \| Categorical (MDD vs HC): secure lower in MDD (27% vs 57%); insecure styles more common in MDD |
| Wilkinson (2010) | Community | PPD | DSM-IV; MCMI-III | EPDS (EPDS > 12) | Current episode | Dimensional (MDD vs HC): secure lower, anxious higher, avoidant lower, fearful higher in MDD \| Categorical (MDD vs HC): secure lower in MDD (26% vs 71%); insecure styles more common in MDD |
| Yotsidi (2024) | Mixed | MDD | DSM-5; MINI | BDI-II | Current episode | Dimensional (MDD only): anxious, avoidant scores reported |
| Zhang (2023) | Community | MDD | DSM-V; Clinical interview | HRSD-17 | Current episode | Dimensional (MDD only): anxious scores reported |

Notes: Reis et al. (2004) BDI scores are for male participants / female participants. MDD = major depressive disorder; PPD = postpartum depression; HC = healthy control. NR = not reported.

**Table S8. Participant characteristics for each study: race/ethnicity, depressive state**

Notes: Reis et al. (2004) BDI scores are for male participants / female participants.

#### Table S9. Quality Assessment

a. **Studies with HC group**

| Paper (1^st^ author) | Selection  (max 4*) | Comparability  (max 2*) | Exposure  (max 3*) | Total Score | Rating |
| --- | --- | --- | --- | --- | --- |
| Aceti 2012 | *** |  | * | 4 | Poor |
| Bauriedl-Schmidt 2017 | *** | ** | ** | 7 | Good |
| Bifulco 2019 | *** |  | * | 4 | Poor |
| Bracht 2022 | ** | ** | ** | 6 | Moderate |
| Celikbas 2022 | *** |  | ** | 5 | Moderate |
| Costa 2009 | ** |  | ** | 4 | Poor |
| Craba 2023 | *** |  | * | 4 | Poor |
| Demıray 2025 | *** | ** | ** | 7 | Good |
| Fuhr 2017 | ** |  | ** | 4 | Poor |
| Ikeda 2014 | ** | ** | *** | 7 | Good |
| Kani 2023 | ** | * | ** | 5 | Moderate |
| Karabekiroglu 2011 | * |  | ** | 3 | Poor |
| Koelkeback 2017 | ** | ** | * | 5 | Moderate |
| König 2026 | *** | ** | ** | 7 | Good |
| Kwon 2017 | *** |  | *** | 6 | Moderate |
| Marazziti 2007 | *** |  | ** | 5 | Moderate |
| Martucci 2021 | *** | * | * | 5 | Moderate |
| McBride 2006 | * |  | ** | 3 | Poor |
| McMahon 2005 | *** | ** | ** | 7 | Good |
| Melnikov 2021 | *** | ** | ** | 7 | Good |
| Murray 2006 | *** | ** | *** | 8 | Good |
| Niolu 2016 | ** | ** | * | 5 | Moderate |
| Nonnenmacher 2016 | *** | * | ** | 6 | Moderate |
| Özaslan 2025 | *** | ** | ** | 7 | Good |
| Ozer 2015 | *** | ** | ** | 7 | Good |
| Paradiso 2011 | *** |  | ** | 5 | Moderate |
| Picardi 2019 | *** |  | ** | 5 | Moderate |
| Reiner 2016 | *** | ** | ** | 7 | Good |
| Reis 2004 | * | * | * | 3 | Poor |
| Santona 2015 | *** | ** | ** | 7 | Good |
| Strecker 2025 | *** | ** | ** | 7 | Good |
| Whiffen 2001 | *** | * | * | 5 | Moderate |
| Wilkinson 2010 | *** |  | ** | 5 | Moderate |

b. **Studies with only MDD samples**

| Paper (1^st^ author) | Selection  (max 5*) | Comparability  (max 2*) | Outcome  (max 3*) | Total Score | Rating |
| --- | --- | --- | --- | --- | --- |
| A-Tjak 2020 | **** |  | ** | 6 | Moderate |
| Bartolomei 2020 | *** | ** | ** | 7 | Good |
| Bernecker 2016 | *** |  | ** | 5 | Moderate |
| Bifulco 2002 | *** | * | *** | 7 | Good |
| Cacciola 2020 | *** |  | ** | 5 | Moderate |
| Cheng 2025 | *** | ** | ** | 7 | Good |
| Cho 2025 | *** |  | ** | 5 | Moderate |
| Conradi 2009 | *** |  | ** | 5 | Moderate |
| Dagnino 2017 | *** | ** | ** | 7 | Good |
| DeFilippo 2002 | *** | ** | ** | 7 | Good |
| Gilbert 2009 | ** |  | ** | 4 | Poor |
| Golshani 2021 | **** |  | ** | 6 | Moderate |
| Grunebaum 2010 | ** | ** | ** | 6 | Moderate |
| Huang 2019 | *** | ** | *** | 8 | Good |
| Huh 2019 | **** | ** | ** | 8 | Good |
| Ihme 2022 | **** |  | ** | 6 | Moderate |
| Levitan 2009 | *** |  | ** | 5 | Moderate |
| Martinez 2023 | *** | ** | ** | 7 | Good |
| Nowak 2026 | *** | ** | ** | 7 | Good |
| Oon-Arom 2019 | ** | ** | ** | 6 | Moderate |
| Reinecke 2001 | **** |  | ** | 6 | Moderate |
| Smith 2012 | *** | ** | ** | 7 | Good |
| Spence 2020 | **** |  | ** | 6 | Moderate |
| Strauss 2006 | **** | * | ** | 7 | Good |
| Troxel 2007 | *** | ** | ** | 7 | Good |
| Watson 2014 | *** |  | ** | 5 | Moderate |
| Yotsidi 2024 | *** |  | ** | 4 | Poor |
| Zhang 2023 | *** | ** | ** | 7 | Good |

#### Supplementary: Attachment findings by instrument

These analyses ask whether the pooled results depend on how attachment was measured. Two of the four attachment styles behaved the same way on every instrument and one did not. Reduced secure attachment and elevated fearful attachment were significant on every measure contributing at least three studies. For elevated avoidant attachment, it was significant on some instruments and absent on others, and scale family was the only moderator to reach significance in the meta-regressions. Anxious attachment was intermediate, significant on the two larger instrument subgroups and in the same direction but not significant on the two smaller ones.

In categorical analyses (Table S10b), reduced secure attachment replicated on all four instruments with at least three contributing studies: RQ (k = 3, OR = 0.30 [0.13, 0.71], p = .006), ECR (k = 6, OR = 0.13 [0.07, 0.22], p < .001), AAI (k = 3, OR = 0.17 [0.08, 0.40], p < .001) and ASI (k = 3, OR = 0.20 [0.11, 0.37], p < .001). Elevated fearful attachment replicated wherever it was measured: RQ (OR = 3.79 [2.23, 6.46], p < .001), ECR (OR = 3.64 [1.20, 11.02], p = .022) and ASI (OR = 3.54 [1.03, 12.23], p = .045). Anxious attachment was significantly elevated on the ECR (OR = 4.03 [2.22, 7.31], p < .001) and the ASI (OR = 2.30 [1.04, 5.09], p = .039), and in the same direction but not significant on the RQ (OR = 2.08 [0.75, 5.73], p = .157) and the AAI (OR = 3.55 [0.76, 16.65], p = .109), where k = 3 and the intervals are wide. Avoidant attachment reached significance only on the ASI (OR = 2.79 [1.07, 7.24], p = .035); it did not on the ECR (OR = 1.63 [0.92, 2.88], p = .092), the AAI (OR = 1.16 [0.62, 2.18], p = .635) or the RQ (OR = 0.60 [0.28, 1.30], p = .197).

In continuous analyses (Table S10a), the ECR-family showed substantial elevations in both anxious (k = 4, g = 0.91 [0.61, 1.21], p < .001) and avoidant attachment (k = 4, g = 0.93 [0.58, 1.29], p < .001). On RQ, secure attachment was reduced (k = 3, g = −0.81 [−1.17, −0.45], p < .001) and fearful attachment elevated (k = 3, g = 0.54 [0.33, 0.74], p < .001), whereas anxious attachment did not reach significance (k = 3, g = 0.43 [−0.05, 0.92], p = .081) and avoidant attachment showed no difference at all (k = 3, g = −0.01 [−0.20, 0.17], p = .897).

In meta-regressions, attachment scale family did not moderate any categorical outcome (Table S13a, all p ≥ .13, with the AAI as the reference category). In continuous meta-regressions (Table S13b, AAS-family as reference) it moderated avoidance alone as the ECR-family differed from the AAS-family by β = +1.28 [+0.14, +2.43], p = .028, the only significant scale-family contrast in either set.

The ECR-family measures avoidance as a continuous dimension of discomfort with closeness and self-reliance, on which a patient may score highly while also scoring highly on anxiety. The RQ and the other measures instead assign each respondent to one of four mutually exclusive categories, in which high avoidance combined with high anxiety is classified as fearful rather than as dismissing-avoidant. Patients who are both anxious and avoidant are therefore counted as avoidant on a dimensional instrument and as fearful on a prototype instrument. The RQ's large fearful elevation (OR = 3.79) alongside its null avoidant result (OR = 0.60) is what that classification rule predicts, and the two findings can be understood as single result rather than as a contradiction. This is one reason the avoidant finding is the least robust of the three insecure patterns in the main analyses.

These subgroup analyses are exploratory. Each rests on three to six studies, the intervals are correspondingly wide, and no adjustment was made for multiple comparisons. They are intended to show where the pooled estimates are, and are not, sensitive to how attachment was measured, rather than to provide instrument-specific estimates in their own right.

#### Table S10a. Continuous Meta-Analysis Scale-Specific Exploratory Subgroup Analyses

| **Scale** | **Attachment style** | **k** | **Hedges' g** | **95% CI** | **p** | **I² (%)** |
| --- | --- | --- | --- | --- | --- | --- |
| ECR-family | Anxious | 4 | 0.91 | [0.61, 1.21] | < .001 | 40.7 |
| ECR-family | Avoidant | 4 | 0.93 | [0.58, 1.29] | < .001 | 57.4 |
| RQ | Secure | 3 | −0.81 | [−1.17, −0.45] | < .001 | 68.4 |
| RQ | Anxious | 3 | 0.43 | [−0.05, 0.92] | .081 | 83.5 |
| RQ | Avoidant | 3 | −0.01 | [−0.20, 0.17] | .897 | 0.0 |
| RQ | Fearful | 3 | 0.54 | [0.33, 0.74] | < .001 | 10.0 |

Scale-specific exploratory subgroup analyses of continuous (dimensional) attachment outcomes. Hedges' g = standardised mean difference (MDD vs healthy controls). ECR-family includes ECR, ECR-R, and ECR-II. Subgroups with fewer than three contributing studies (e.g., RQ for secure attachment in studies that did not report it) are omitted. The ECR-family group reports anxious and avoidant subscales only; secure and fearful were not consistently reported on the ECR-family two-factor model.

#### Table S10b. Categorical Meta-Analysis Scale-Specific Exploratory Subgroup Analyses

| **Scale** | **Attachment style** | **k** | **Pooled OR** | **95% CI** | **p** | **I² (%)** |
| --- | --- | --- | --- | --- | --- | --- |
| RQ | Secure | 3 | 0.30 | [0.13, 0.71] | .006 | 67.6 |
| RQ | Anxious | 3 | 2.08 | [0.75, 5.73] | .157 | 62.9 |
| RQ | Avoidant | 3 | 0.60 | [0.28, 1.30] | .197 | 12.2 |
| RQ | Fearful | 3 | 3.79 | [2.23, 6.46] | < .001 | 0.0 |
| ECR | Secure | 6 | 0.13 | [0.07, 0.22] | < .001 | 42.7 |
| ECR | Anxious | 4 | 4.03 | [2.22, 7.31] | < .001 | 0.0 |
| ECR | Avoidant | 4 | 1.63 | [0.92, 2.88] | .092 | 23.2 |
| ECR | Fearful | 4 | 3.64 | [1.20, 11.02] | .022 | 64.7 |
| AAI | Secure | 3 | 0.17 | [0.08, 0.40] | < .001 | 47.6 |
| AAI | Anxious | 3 | 3.55 | [0.76, 16.65] | .109 | 73.0 |
| AAI | Avoidant | 3 | 1.16 | [0.62, 2.18] | .635 | 12.6 |
| ASI | Secure | 3 | 0.20 | [0.11, 0.37] | < .001 | 0.0 |
| ASI | Anxious | 3 | 2.30 | [1.04, 5.09] | .039 | 0.0 |
| ASI | Avoidant | 3 | 2.79 | [1.07, 7.24] | .035 | 12.1 |
| ASI | Fearful | 3 | 3.54 | [1.03, 12.23] | .045 | 38.9 |

Scale-specific exploratory subgroup analyses of categorical (typological) attachment outcomes. Pooled OR = pooled odds ratio for the attachment style in MDD relative to healthy controls. The three additional studies identified in the updated search used the RSQ (Strecker 2025; combined with Çelikbaş 2022 yielding k = 2) and the AASS (Demıray 2025; k = 1), which fell below the predetermined minimum of three contributing studies and were therefore not analysed as separate scale-specific subgroups.

#### Table S11a. Categorical Meta-Analysis MDD-Only Subgroup

| **Attachment style** | **k** | **Pooled OR** | **95% CI** | **z** | **p** | **I² (%)** |
| --- | --- | --- | --- | --- | --- | --- |
| Secure | 14 | 0.19 | [0.13, 0.28] | −8.33 | < .001 | 58.5 |
| Anxious | 11 | 2.20 | [1.44, 3.37] | 3.65 | < .001 | 38.4 |
| Avoidant | 11 | 1.42 | [1.02, 1.99] | 2.06 | .039 | 26.4 |
| Fearful | 9 | 2.77 | [1.71, 4.47] | 4.15 | < .001 | 38.2 |

OR = odds ratio for the attachment style in patients with major depressive disorder (MDD) relative to healthy controls; CI = confidence interval; z = standard normal test statistic; I² = heterogeneity. Pooled using REML random-effects meta-analysis with Haldane–Anscombe continuity correction for zero cells.

**Table S11b. Categorical Meta-Analysis PPD-Only Subgroup**

| **Attachment style** | **k** | **Pooled OR** | **95% CI** | **z** | **p** | **I² (%)** |
| --- | --- | --- | --- | --- | --- | --- |
| Secure | 7 | 0.19 | [0.13, 0.29] | −8.28 | < .001 | 0.0 |
| Anxious | 5 | 3.17 | [1.28, 7.85] | 2.49 | .013 | 53.0 |
| Avoidant | 5 | 1.28 | [0.53, 3.07] | 0.56 | .579 | 50.3 |
| Fearful | 3 | 3.80 | [1.65, 8.80] | 3.12 | .002 | 34.3 |

Postpartum depression (PPD) subgroup. OR = odds ratio for the attachment style in patients with PPD relative to healthy controls. CI = confidence interval; z = standard normal test statistic; I² = heterogeneity. Pooled using REML random-effects meta-analysis with Haldane–Anscombe continuity correction for zero cells.

**Table S12a. Continuous Meta-Analysis MDD-Only Subgroup**

| **Attachment style** | **k** | **Hedges’ g** | **95% CI** | **z** | **p** | **I² (%)** |
| --- | --- | --- | --- | --- | --- | --- |
| Secure | 6 | −0.35 | [−0.62, −0.08] | −2.55 | .011 | 80.4 |
| Anxious | 12 | +0.59 | [+0.35, +0.84] | 4.73 | < .001 | 83.0 |
| Avoidant | 12 | +0.36 | [−0.00, +0.71] | 1.95 | .051 | 92.5 |
| Fearful | 4 | +0.27 | [−0.15, +0.69] | 1.30 | .195 | 88.2 |

MDD-only continuous subgroup analysis. Hedges' g = standardised mean difference (MDD vs healthy controls) with small-sample bias correction. REML random-effects meta-analysis on the standardised mean difference scale.

**Table S12b. Continuous Meta-Analysis PPD-Only Subgroup**

| **Attachment style** | **k** | **Hedges’ g** | **95% CI** | **z** | **p** | **I² (%)** |
| --- | --- | --- | --- | --- | --- | --- |
| Secure | 1 | — | — | — | — | — |
| Anxious | 3 | +0.80 | [+0.47, +1.12] | 4.81 | < .001 | 41.8 |
| Avoidant | 3 | +0.62 | [−0.12, +1.36] | 1.63 | .103 | 88.8 |
| Fearful | 1 | — | — | — | — | — |

PPD-only continuous subgroup analysis. Only two PPD case-control studies (Wilkinson 2010, Martucci 2021) contributed to this subgroup, severely limiting statistical power and inference. Secure and fearful attachment could not be summarised (k = 1).

**Table S13a. Univariate Meta-Regression of Moderators on Categorical Attachment Outcomes (log OR)**

| **Outcome** | **Moderator** | **k** | **β [95% CI]** | **p** | **Residual I²** |
| --- | --- | --- | --- | --- | --- |
| Secure | Mean age (MDD) | 17 | +0.059 [-0.266, +0.385] | 0.721 | 53.4% |
| Secure | % female (MDD) | 20 | +0.063 [-0.253, +0.378] | 0.698 | 50.7% |
| Secure | NOS quality score | 21 | -0.010 [-0.252, +0.232] | 0.936 | 47.1% |
| Secure | Episode status (current vs range/remitted) | 21 | -0.153 [-0.723, +0.416] | 0.597 | 45.8% |
| Secure | Scale family: AAS-family_vs_AAI | 19 | +0.195 [-0.899, +1.289] | 0.727 | 43.5% |
| Secure | Scale family: ASI_vs_AAI | 19 | +0.143 [-0.901, +1.187] | 0.788 | 43.5% |
| Secure | Scale family: ECR-family_vs_AAI | 19 | -0.324 [-1.227, +0.579] | 0.482 | 43.5% |
| Secure | Scale family: RQ/RSQ_vs_AAI | 19 | +0.602 [-0.277, +1.482] | 0.179 | 43.5% |
| Preoccupied | Mean age (MDD) | 13 | +0.080 [-0.372, +0.532] | 0.728 | 48.0% |
| Preoccupied | % female (MDD) | 16 | +0.037 [-0.363, +0.438] | 0.855 | 50.1% |
| Preoccupied | NOS quality score | 16 | +0.084 [-0.334, +0.503] | 0.693 | 47.3% |
| Preoccupied | Episode status (current vs range/remitted) | 16 | -0.085 [-0.965, +0.796] | 0.851 | 50.4% |
| Preoccupied | Scale family: AAS-family_vs_AAI | 16 | -0.077 [-1.610, +1.457] | 0.922 | 41.7% |
| Preoccupied | Scale family: ASI_vs_AAI | 16 | -0.455 [-1.811, +0.901] | 0.511 | 41.7% |
| Preoccupied | Scale family: ECR-family_vs_AAI | 16 | +0.214 [-0.978, +1.406] | 0.725 | 41.7% |
| Preoccupied | Scale family: RQ/RSQ_vs_AAI | 16 | -0.835 [-1.924, +0.254] | 0.133 | 41.7% |
| Dismissive | Mean age (MDD) | 13 | -0.121 [-0.432, +0.190] | 0.445 | 30.5% |
| Dismissive | % female (MDD) | 16 | -0.270 [-0.524, -0.015] | 0.038 | 4.4% |
| Dismissive | NOS quality score | 16 | -0.003 [-0.293, +0.287] | 0.984 | 27.0% |
| Dismissive | Episode status (current vs range/remitted) | 16 | +0.195 [-0.413, +0.803] | 0.529 | 18.2% |
| Dismissive | Scale family: AAS-family_vs_AAI | 16 | +0.351 [-0.869, +1.570] | 0.573 | 39.7% |
| Dismissive | Scale family: ASI_vs_AAI | 16 | +0.852 [-0.359, +2.062] | 0.168 | 39.7% |
| Dismissive | Scale family: ECR-family_vs_AAI | 16 | +0.347 [-0.591, +1.286] | 0.468 | 39.7% |
| Dismissive | Scale family: RQ/RSQ_vs_AAI | 16 | -0.099 [-1.009, +0.812] | 0.832 | 39.7% |
| Fearful | Mean age (MDD) | 11 | -0.080 [-0.485, +0.325] | 0.699 | 38.3% |
| Fearful | % female (MDD) | 12 | +0.286 [-0.007, +0.578] | 0.055 | 0.0% |
| Fearful | NOS quality score | 12 | -0.103 [-0.563, +0.356] | 0.659 | 39.3% |
| Fearful | Episode status (current vs range/remitted) | 12 | +0.134 [-0.785, +1.052] | 0.775 | 34.7% |
| Fearful | Scale family: ECR-family_vs_ASI | 12 | -0.092 [-1.439, +1.256] | 0.894 | 47.1% |
| Fearful | Scale family: RQ/RSQ_vs_ASI | 12 | -0.182 [-1.413, +1.050] | 0.772 | 47.1% |

Note. Univariate REML meta-regression models for each moderator on each categorical attachment outcome (log odds ratio). Coefficients (β) reflect the change in log-OR per 1-unit increase in the continuous moderator (centred at the sample mean) or relative to the reference category (for scale family). k = number of contributing studies; asterisk (*) flags models with k < 4, which should be interpreted with caution. Residual I² = between-study heterogeneity remaining after adjustment for the moderator. CI = confidence interval. Reference category for scale family is the Adult Attachment Interview (AAI). ECR = Experiences in Close Relationships; AAS = Adult Attachment Style scale; ASI = Attachment Style Interview; RQ/RSQ = Relationship Questionnaire / Relationship Style Questionnaire.

**Table S13b. Univariate Meta-Regression of Moderators on Continuous Attachment Outcomes (Hedges’ g)**

| **Outcome** | **Moderator** | **k** | **β [95% CI]** | **p** | **Residual I²** |
| --- | --- | --- | --- | --- | --- |
| Secure | Mean age (MDD) | 5 | +0.295 [-0.109, +0.699] | 0.153 | 90.7% |
| Secure | % female (MDD) | 6 | -0.381 [-0.595, -0.168] | < .001 | 66.5% |
| Secure | NOS quality score | 7 | -0.056 [-0.400, +0.287] | 0.748 | 90.6% |
| Secure | Episode status (current vs range/remitted) | 7 | -0.432 [-1.010, +0.146] | 0.143 | 86.4% |
| Secure | Scale family: Other_vs_AAS-family | 7 | +0.241 [-0.932, +1.414] | 0.687 | 93.1% |
| Secure | Scale family: RQ/RSQ_vs_AAS-family | 7 | -0.094 [-1.176, +0.988] | 0.865 | 93.1% |
| Anxious | Mean age (MDD) | 12 | +0.073 [-0.182, +0.329] | 0.573 | 81.4% |
| Anxious | % female (MDD) | 13 | -0.072 [-0.333, +0.189] | 0.588 | 84.1% |
| Anxious | NOS quality score | 15 | +0.235 [+0.043, +0.427] | 0.017 | 77.9% |
| Anxious | Episode status (current vs range/remitted) | 15 | -0.064 [-0.552, +0.424] | 0.796 | 85.4% |
| Anxious | Scale family: ECR-family_vs_AAS-family | 15 | -0.062 [-0.744, +0.620] | 0.858 | 85.0% |
| Anxious | Scale family: Other_vs_AAS-family | 15 | -0.506 [-1.206, +0.194] | 0.156 | 85.0% |
| Anxious | Scale family: RQ/RSQ_vs_AAS-family | 15 | -0.509 [-1.144, +0.127] | 0.117 | 85.0% |
| Avoidant | Mean age (MDD) | 12 | +0.119 [-0.310, +0.547] | 0.588 | 94.2% |
| Avoidant | % female (MDD) | 13 | +0.133 [-0.299, +0.564] | 0.547 | 95.0% |
| Avoidant | NOS quality score | 15 | +0.155 [-0.180, +0.490] | 0.364 | 94.2% |
| Avoidant | Episode status (current vs range/remitted) | 15 | -0.036 [-0.803, +0.731] | 0.926 | 94.6% |
| Avoidant | Scale family: ECR-family_vs_AAS-family | 15 | +1.284 [+0.136, +2.432] | 0.028 | 94.2% |
| Avoidant | Scale family: Other_vs_AAS-family | 15 | +0.787 [-0.405, +1.979] | 0.195 | 94.2% |
| Avoidant | Scale family: RQ/RSQ_vs_AAS-family | 15 | +0.535 [-0.536, +1.606] | 0.327 | 94.2% |
| Fearful | Mean age (MDD) | 3 * | -0.060 [-0.317, +0.196] | 0.644 | 53.2% |
| Fearful | % female (MDD) | 4 | +0.275 [-0.178, +0.728] | 0.234 | 91.7% |
| Fearful | NOS quality score | 5 | +0.271 [-0.028, +0.570] | 0.075 | 84.2% |
| Fearful | Episode status (current vs range/remitted) | 5 | -0.313 [-1.107, +0.482] | 0.440 | 90.8% |

Note. Univariate REML meta-regression models for each moderator on each continuous (dimensional) attachment outcome (standardised mean difference). β = change in Hedges' g per 1-unit increase in the continuous moderator (centred) or relative to the reference category (AAS-family for scale family). k = number of contributing studies; asterisk (*) flags models with k < 4, which should be interpreted with caution. AAS-family is the reference category for the scale-family moderator in the continuous analyses.

#### Table S14. Pooled Prevalence of Attachment Styles in MDD/PPD

| **Attachment style** | **k** | **Events/N** | **Pooled prevalence, % [95% CI]** | **Sensitivity (IV-REML), % [95% CI]** | **I² (%)** | **95% PI** |
| --- | --- | --- | --- | --- | --- | --- |
| Secure | 29 | 470/1905 | 24.1 [19.8, 28.9] | 24.9 [20.7, 29.7] | 78.6 | 8.6–51.5 |
| Anxious / preoccupied | 25 | 558/1753 | 23.0 [17.8, 29.2] | 24.4 [19.0, 30.9] | 91.5 | 5.8–59.0 |
| Avoidant / dismissive | 25 | 444/1753 | 23.7 [18.0, 30.5] | 25.4 [19.8, 32.0] | 84.1 | 5.2–63.6 |
| Fearful | 20 | 317/1178 | 26.9 [21.9, 32.5] | 27.7 [22.7, 33.3] | 72.2 | 10.9–52.5 |

Pooled prevalence of each attachment style within clinically diagnosed MDD/PPD, estimated by random-effects single-arm meta-analysis of proportions using a random-intercept logistic model (binomial–normal generalised linear mixed model) on the logit scale and back-transformed to the proportion scale. All eligible MDD/PPD samples reporting mutually exclusive categorical classifications are included, irrespective of whether the study had a healthy-control group. IV-REML = inverse-variance random-effects model with REML estimation on the logit scale, fitted as a sensitivity analysis. Between-study variance on the logit scale (τ²) was 0.332 (secure), 0.551 (anxious), 0.665 (avoidant), 0.256 (fearful); I² = heterogeneity. The 95% prediction interval describes the range of true prevalences expected across settings.

#### Table S15. Prevalence of Attachment Styles, MDD versus PPD Subgroups

| **Attachment style** | **k** | **MDD prevalence, % [95% CI]** | **k** | **PPD prevalence, % [95% CI]** | **Q between** | **p** |
| --- | --- | --- | --- | --- | --- | --- |
| Secure | 21 | 21.3 [16.7, 26.9] | 8 | 31.9 [26.3, 38.2] | 6.91 | .009 |
| Anxious / preoccupied | 17 | 25.4 [19.3, 32.5] | 8 | 19.2 [11.0, 31.5] | 0.87 | .350 |
| Avoidant / dismissive | 17 | 26.9 [20.6, 34.3] | 8 | 16.6 [7.9, 31.7] | 1.74 | .187 |
| Fearful | 14 | 26.3 [21.0, 32.2] | 6 | 28.2 [17.5, 42.2] | 0.08 | .775 |

Prevalence of each attachment style estimated separately in MDD and postpartum depression (PPD) samples, using the same random-intercept logistic model as Table S14. Subgroup differences were tested by Q between on the logit scale with 1 degree of freedom; k = number of contributing samples in each subgroup. Residual heterogeneity (I²) was 83.0% (MDD) and 14.9% (PPD) for secure; 93.7% (MDD) and 69.6% (PPD) for anxious; 87.0% (MDD) and 74.9% (PPD) for avoidant; 73.1% (MDD) and 69.1% (PPD) for fearful. Secure attachment was significantly more common in PPD than in MDD samples; the three insecure styles did not differ significantly between the two contexts.

**Figure S1. Funnel plots of studies with continuous and categorical outcomes in four attachment types**

a. **Funnel plots of continuous outcomes in secure attachment**

b. **Funnel plots of continuous outcomes in anxious attachment**

c. **Funnel plots of continuous outcomes in avoidant attachment**

d. **Funnel plots of continuous outcomes in fearful attachment**

e. **Funnel plots of categorical outcomes in secure attachment**

f. **Funnel plots of categorical outcomes in anxious attachment**

g. **Funnel plots of categorical outcomes in avoidant attachment**

h. **Funnel plots of categorical outcomes in fearful attachment**

**Figure S2. MDD-Only Categorical Forest Plots**

Pooled estimates and confidence intervals were obtained using REML random-effects meta-analysis. Studies are sorted chronologically; the dark orange diamond shows the pooled odds ratio.

**Figure S2a. Secure attachment MDD only**

**Figure S2b. Anxious attachment MDD only**

**Figure S2c. Avoidant attachment MDD only**

**Figure S2d. Fearful attachment MDD only**

**Figure S3. PPD-Only Categorical Forest Plots**

Pooled estimates and confidence intervals were obtained using REML random-effects meta-analysis. Studies are sorted chronologically; the dark orange diamond shows the pooled odds ratio.

**Figure S3a. Secure attachment PPD only**

**Figure S3b. Anxious attachment PPD only**

**Figure S3c. Avoidant attachment PPD only**

**Figure S3d. Fearful attachment PPD only**

**Figure S4. Pooled prevalence of attachment styles in clinically diagnosed MDD/PPD**

Random-effects single-arm meta-analyses of the proportion of patients classified into each attachment style, pooled on the logit scale using a random-intercept logistic model: (a) secure (k = 29), (b) anxious/preoccupied (k = 25), (c) avoidant/dismissive (k = 25), (d) fearful (k = 20). Green squares show individual study proportions with exact (Clopper–Pearson) 95% confidence intervals, sized in proportion to their random-effects weight; the orange diamond shows the pooled prevalence with its 95% confidence interval. All eligible MDD/PPD samples reporting mutually exclusive categorical classifications are included, irrespective of whether the study had a healthy-control group. Heterogeneity (τ², Cochran’s Q, I²) and the 95% prediction interval are reported below each panel.

a. Secure Attachment (Prevalence)

b. Anxious / Preoccupied Attachment (Prevalence)

c. Avoidant / Dismissive Attachment (Prevalence)

d. Fearful Attachment (Prevalence)
